# Molecular Pathogenesis of EBV Susceptibility in XLP as Revealed by Analysis of Female Carriers with Heterozygous Expression of SAP

**DOI:** 10.1371/journal.pbio.1001187

**Published:** 2011-11-01

**Authors:** Umaimainthan Palendira, Carol Low, Anna Chan, Andrew D. Hislop, Edwin Ho, Tri Giang Phan, Elissa Deenick, Matthew C. Cook, D. Sean Riminton, Sharon Choo, Richard Loh, Frank Alvaro, Claire Booth, H. Bobby Gaspar, Alessandro Moretta, Rajiv Khanna, Alan B. Rickinson, Stuart G. Tangye

**Affiliations:** 1 Garvan Institute of Medical Research, Darlinghurst, New South Wales, Australia; 2 St. Vincent's Clinical School, University of New South Wales, Darlinghurst, New South Wales, Australia; 3 School of Cancer Sciences and MRC Centre for Immune Regulation, University of Birmingham, Edgbaston, United Kingdom; 4 Australian National University Medical School, Canberra, Australian Capital Territory, Australia; 5 John Curtin School of Medical Research, Canberra, Australian Capital Territory, Australia; 6 Department of Immunology, Canberra Hospital, Canberra, Australian Capital Territory, Australia; 7 Department of Immunology, Concord Hospital, Sydney, New South Wales, Australia; 8 Department of Allergy and Immunology, Royal Children's Hospital Melbourne, Melbourne, Victoria, Australia; 9 Department of Clinical Immunology, Princess Margaret Hospital for Children, Perth, Western Australia, Australia; 10 Pediatric Hematology, John Hunter Hospital, Newcastle, New South Wales, Australia; 11 Centre for Immunodeficiency, Molecular Immunology Unit, UCL Institute of Child Health, London, United Kingdom; 12 Dipartimento di Medicina Sperimentale, Università di Genova, Genova, Italy; 13 Tumour Immunology Laboratory, Division of Immunology, Queensland Institute of Medical Research, Brisbane, Queensland, Australia; University of Wisconsin-Madison, United States of America

## Abstract

X-linked lymphoproliferative disease (XLP) is a primary immunodeficiency caused by mutations in *SH2D1A* which encodes SAP. SAP functions in signalling pathways elicited by the SLAM family of leukocyte receptors. A defining feature of XLP is exquisite sensitivity to infection with EBV, a B-lymphotropic virus, but not other viruses. Although previous studies have identified defects in lymphocytes from XLP patients, the unique role of SAP in controlling EBV infection remains unresolved. We describe a novel approach to this question using female XLP carriers who, due to random X-inactivation, contain both SAP^+^ and SAP^−^ cells. This represents the human equivalent of a mixed bone marrow chimera in mice. While memory CD8^+^ T cells specific for CMV and influenza were distributed across SAP^+^ and SAP^−^ populations, EBV-specific cells were exclusively SAP^+^. The preferential recruitment of SAP^+^ cells by EBV reflected the tropism of EBV for B cells, and the requirement for SAP expression in CD8^+^ T cells for them to respond to Ag-presentation by B cells, but not other cell types. The inability of SAP^−^ clones to respond to Ag-presenting B cells was overcome by blocking the SLAM receptors NTB-A and 2B4, while ectopic expression of NTB-A on fibroblasts inhibited cytotoxicity of SAP^−^ CD8^+^ T cells, thereby demonstrating that SLAM receptors acquire inhibitory function in the absence of SAP. The innovative XLP carrier model allowed us to unravel the mechanisms underlying the unique susceptibility of XLP patients to EBV infection in the absence of a relevant animal model. We found that this reflected the nature of the Ag-presenting cell, rather than EBV itself. Our data also identified a pathological signalling pathway that could be targeted to treat patients with severe EBV infection. This system may allow the study of other human diseases where heterozygous gene expression from random X-chromosome inactivation can be exploited.

## Abstract 2

Analysis of females carriers of the X-linked lymphoproliferative (XLP) trait reveals the mechanism underlying exquisite sensitivity of XLP patients to often-fatal infection with the normally innocuous Epstein-Barr virus.

## Author Summary

X-linked lymphoproliferative disease (XLP) is an immunodeficiency caused by mutations in the SH2D1A gene, which encodes a cytoplasmic component, SAP involved in a signalling pathway in certain populations of immune cells. The Achilles' heel in XLP is extreme sensitivity to Epstein-Barr virus (EBV) infection. Although EBV infection in normal individuals is generally innocuous, in XLP it can be fatal. Strikingly, individuals with XLP do not display this same vulnerability to other viruses, and here we investigate what immune defects underlie this specific susceptibility. We developed a system to examine the behaviour of immune cells that are identical with the exception of whether or not they have a functional SH2D1A gene. This approach uses human female carriers of XLP (one of their X chromosomes carries the mutation). Following the process of X-chromosome inactivation in female cells, which is random, individuals harbour T cells that express the normal SH2D1A gene as well as cells that express the mutated version. We found that SAP-deficient CD8^+^ T cells fail to be activated by antigen-presenting B cells, but are activated by other antigen-presenting cell types. Since EBV selectively infects B cells, the exquisite sensitivity in XLP to EBV infection results from the ability of the virus to sequester itself in B cells, which can only induce a cytotoxic T cell response in SAP-sufficient cells. Thus, the functional defect in SAP-deficient CD8^+^ T cells does not relate to a specific virus but rather to the nature of the target cell presenting viral epitopes.

## Introduction

X-linked lymphoproliferative disease (XLP) is an inherited primary immunodeficiency caused by mutations in *SH2D1A*, which encodes the cytoplasmic adaptor protein SLAM-associated protein (SAP) [1]–[3]. SAP functions as an adaptor protein by associating with members of the SLAM family of surface receptors—SLAM (CD150), 2B4, NTBA, CD84, CD229, and possibly CRACC [4]–[7]—that are expressed on a variety of hemopoietic cells. A defining characteristic of XLP is extreme sensitivity to infection with EBV (reviewed in [7]–[9]). Thus, in contrast to infection of healthy individuals, which is self-limiting, exposure of XLP patients to EBV induces a vigorous and uncontrolled immune response involving polyclonally activated leukocytes. Despite such immune activation, XLP patients fail to control EBV infection, which results in severe and often-fatal fulminant infectious mononucleosis [7]–[9]. XLP patients who survive primary EBV infection can develop hypogammaglobulinemia and B-cell lymphoma, although exposure to EBV is not a prerequisite for these clinical manifestations [8],[9]. Strikingly, XLP patients do not display the same degree of vulnerability towards other herpes viruses—herpes simplex virus, cytomegalovirus (CMV), varicella zoster—which can cause life-threatening infections in individuals with other immunodeficiencies [10]. This highlights the unique role of EBV in the pathogenesis of XLP, and the critical—albeit undefined—role of SAP in anti-EBV immunity.

XLP is associated with a diverse range of lymphocyte defects including abolished NKT cell development [11],[12], compromised humoral immunity [13]–[15], and impaired functions of CD4^+^ T cells [13],[16]–[18], CD8^+^ T cells [19],[20], and NK cells [21]–[27]. This reflects the involvement of SAP in multiple signalling pathways. Given the complexity of the immunological abnormalities in XLP patients, it is unclear which of them underlies their unique susceptibility to EBV. While the defective response of NK cells following engagement of 2B4 or NTB-A may contribute to the susceptibility to EBV in XLP [22],[24],[26],[27], it is unlikely to be the predominant cause since a deficiency in either the absolute number of NK cells or NK cell cytotoxicity in the presence of intact T cell development and function in humans is associated with more generalised susceptibility to multiple viruses (reviewed in [28]). Similarly, while NKT cells may have a role in anti-viral immunity, the impact of an NKT cell deficiency on EBV sensitivity in XLP is unclear because patients with other immunodeficiencies have also been reported to lack NKT cells, yet they do not develop fulminant infectious mononucleosis [29]–[31]. Lastly, while several previous studies have investigated the function of CD8^+^ T cells in XLP [19],[20],[32], it is difficult to separate direct effects of SAP deficiency in these cells from indirect effects that may result from lack of “help” from either functionally impaired SAP-deficient CD4^+^ T cells or NK cells, or the absence of NKT cells, all of which can promote CD8^+^ T cell responses [33]–[36]. Furthermore, these studies of SAP-deficient CD8^+^ T cells have not provided an explanation as to why XLP patients are so vulnerable to infection with EBV, but not with other pathogens.

In addition to these issues, delineating the EBV-specific defect in XLP has been hindered by the lack of an appropriate experimental model. Thus, while SAP-deficient mice have proved key to elucidating mechanisms underlying some of the immunological defects in XLP [4],[7],[9], they cannot directly address the question of EBV susceptibility because neither EBV nor its close relatives in other primates infect mice, and no mouse virus can reproduce EBV's biology or its strictly B-lymphotropic means of persistence [37]. The question of EBV pathogenesis therefore can only be answered using a human model in which SAP-deficient immune cells develop in an otherwise intact immune system. Fortuitously, female carriers of XLP are healthy [38] and harbour both SAP-positive and SAP-negative T cells through random inactivation of the X-chromosome [11].

Here we demonstrate that such XLP carriers provide an ideal model for elucidating the role of SAP in anti-viral immune responses in humans. XLP carriers were shown to contain both SAP^+^ and SAP^−^ T cells, which allowed us to determined which virus-specific responses were dependent on SAP. While both SAP^+^ and SAP^−^ CMV or influenza-specific memory CD8^+^ T cells were able to respond to their cognate peptides, EBV-specific memory CD8^+^ T cells were exclusively restricted to the SAP^+^ population, revealing a specific requirement for SAP in anti-EBV immunity. Further analysis of the response of SAP^−^ CD8^+^ T cells to different Ag-presenting cells (APC) showed that SAP is required for B cell-mediated CD8^+^ T cell responses but not for responses induced by other APCs. Our studies further demonstrated that an important function of SAP was to prevent the delivery of inhibitory signals downstream of SLAM family receptors on CD8^+^ T cells following interaction with their ligands on target B-cells. These data provide compelling evidence that the unique susceptibility to EBV infection in XLP patients is due to the inability of SAP^−^ CD8^+^ T cells to respond to Ag-presenting B cells due to inhibitory signalling mediated by SLAM family receptors, rather than an inability to recognise and respond to EBV Ags.

## Results

### Lymphocyte Defects Characteristic of XLP Patients Are Not Present in XLP Carriers

**Figure 1:**
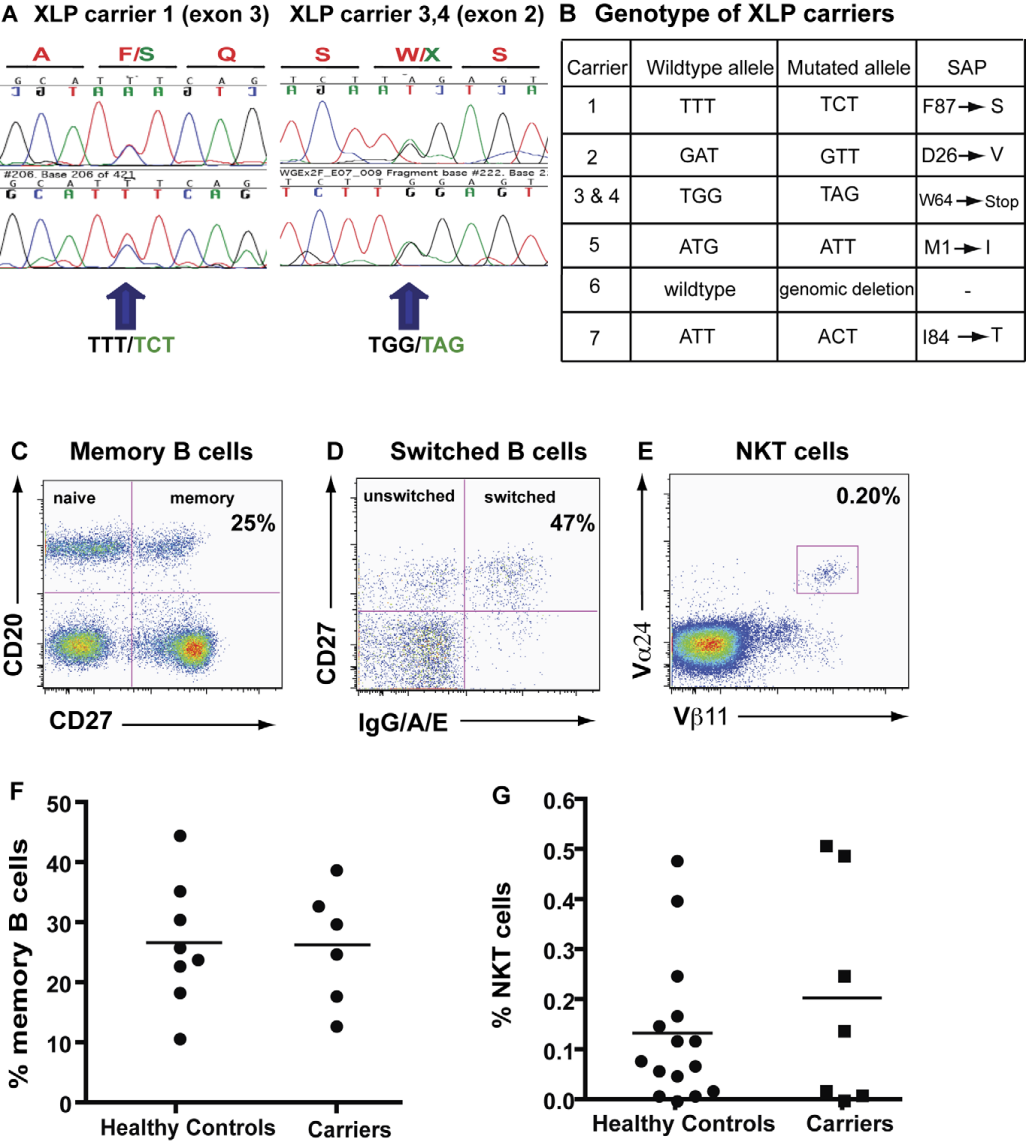
Immune features of heterozygote carriers of XLP. (A) Forward (upper) and reverse (lower) genomic DNA sequences of affected exons in three representative female XLP carriers. (B) The wild-type and mutated alleles and resulting amino acid changes in the seven XLP carriers used in this study. (C–E) PBMCs from XLP carriers were labelled with mAb against CD20, CD27, and IgG/A/E or CD3, TCRVβ11, and TCR Vα24. The frequency of: (C, F) B cells expressing CD27 (i.e., memory cells); (D) memory B cells expressing isotype switched Ig; and (E, G) NKT cells were then determined. The values depicted in dot plots in (C), (D), and (E) correspond to the mean frequency of total memory B cells, isotype switched memory B cells, and NKT cells, respectively. Reference values for healthy controls have been previously published [15],[16],[29].

We analysed seven female carriers of XLP, each of whom was confirmed as heterozygous at the *SH2D1A* locus by sequencing genomic DNA (Figure 1A,B). Analysis of lymphocyte subsets revealed that these carriers, unlike XLP patients [11],[15],[16], had normal frequencies of total and isotype switched memory B cells (Figure 1C,D,F) and NKT cells (Figure 1E,G). The proportions of memory CD8^+^ and CD4^+^ T cells were also within the range of healthy controls (unpublished data). This is consistent with XLP carriers being asymptomatic and lacking evidence of any obvious deficiency in anti-viral immune responses, including against EBV [38],[39].

### XLP Carriers Have Both SAP^+^ and SAP^−^ CD8^+^ T Cells

**Figure 2:**
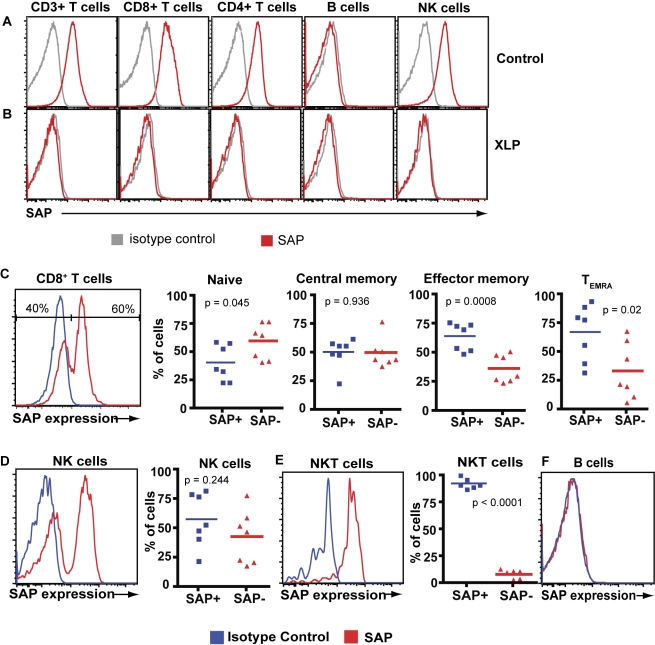
Heterozygous SAP expression in T cells and NK cells from XLP carriers. (A, B) PBMCs from a healthy donor (A) or an XLP patient (B) were incubated with mAb against CD3, CD4, CD8, CD56, and CD20. The cells were then fixed and permeabilised and labelled with an isotype control (grey histogram) or anti-SAP (red histogram) mAb. Expression of SAP in CD3^+^ T cells, CD8^+^ and CD4^+^ T cells, B cells (CD20^+^), and NK (CD3^−^CD56^+^) cells was then determined. (C–F) PBMCs from XLP carriers were labelled with mAb specific for CD3, CD8, CD45RA, CCR7, CD56, TCRVβ11, TCRVα24, or CD20. The cells were then fixed and permeabilised and incubated with isotype control (blue histogram) or anti-SAP mAb (red histogram). SAP expression and the frequency of SAP^−^ and SAP^+^ cells was determined for: (C) total CD8^+^ T cells, and subsets of naïve (CD45RA^+^CCR7^+^), central memory (CD45RA^−^CCR7^+^), effector memory (CD45RA^−^CCR7^−^), or T_EMRA_ (CD45RA^+^CCR7^−^) cells; (D) NK cells (CD3^−^CD56^+^); (E) NKT cells (CD3^+^TCRVβ11^+^TCRVα24^+^); and (F) B cells (CD20^+^).

Intracellular flow cytometric analysis using a SAP-specific monoclonal antibody (mAb) enabled us to identify SAP expression in different cell populations. SAP was expressed in CD4^+^ T cells, CD8^+^ T cells, and NK cells from normal donors (Figure 2A), but not in the same lymphocyte populations obtained from XLP patients (Figure 2B). Using this approach we confirmed heterozygous SAP expression (i.e., 40%–60% of the cells being SAP^+/−^) within the T and NK cell compartments of XLP carriers (Figure 2C,D). There was no significant difference in the frequency of CD8^+^ central memory (CD45RA^−^CCR7^+^) T cells (Figure 2C) or NK cells (Figure 2D) that were SAP^−^ or SAP^+^. However, significantly more naïve CD8^+^ T cells were SAP^−^ (*p* = 0.045), whereas more effector memory (CD45RA^−^CCR7^−^) and T_EMRA_ (effector memory cells expressing CD45RA) cells were SAP^+^ (Figure 2C). The greater frequency of SAP^−^ cells in the naïve compartment would be consistent with proposed functions for SAP in negatively regulating T cell responses in mice in vivo [40],[41] and in promoting apoptosis of human cells in vitro [42],[43]. In contrast to T and NK cells, >90% of NKT cells in XLP carriers were SAP^+^ (Figure 2E), consistent with the absolute requirement of SAP for their development [11],[12]. SAP was not detected in human B cells (Figure 2A,F) [15], supporting the concept that intrinsic defects in T cells, NK cells, and NKT cells, rather than B cells, are responsible for the XLP phenotype.

### EBV-Specific Cells Are Largely SAP^+^ While CMV and Flu-Specific Cells Are SAP^+^ or SAP^−^

**Figure 3:**
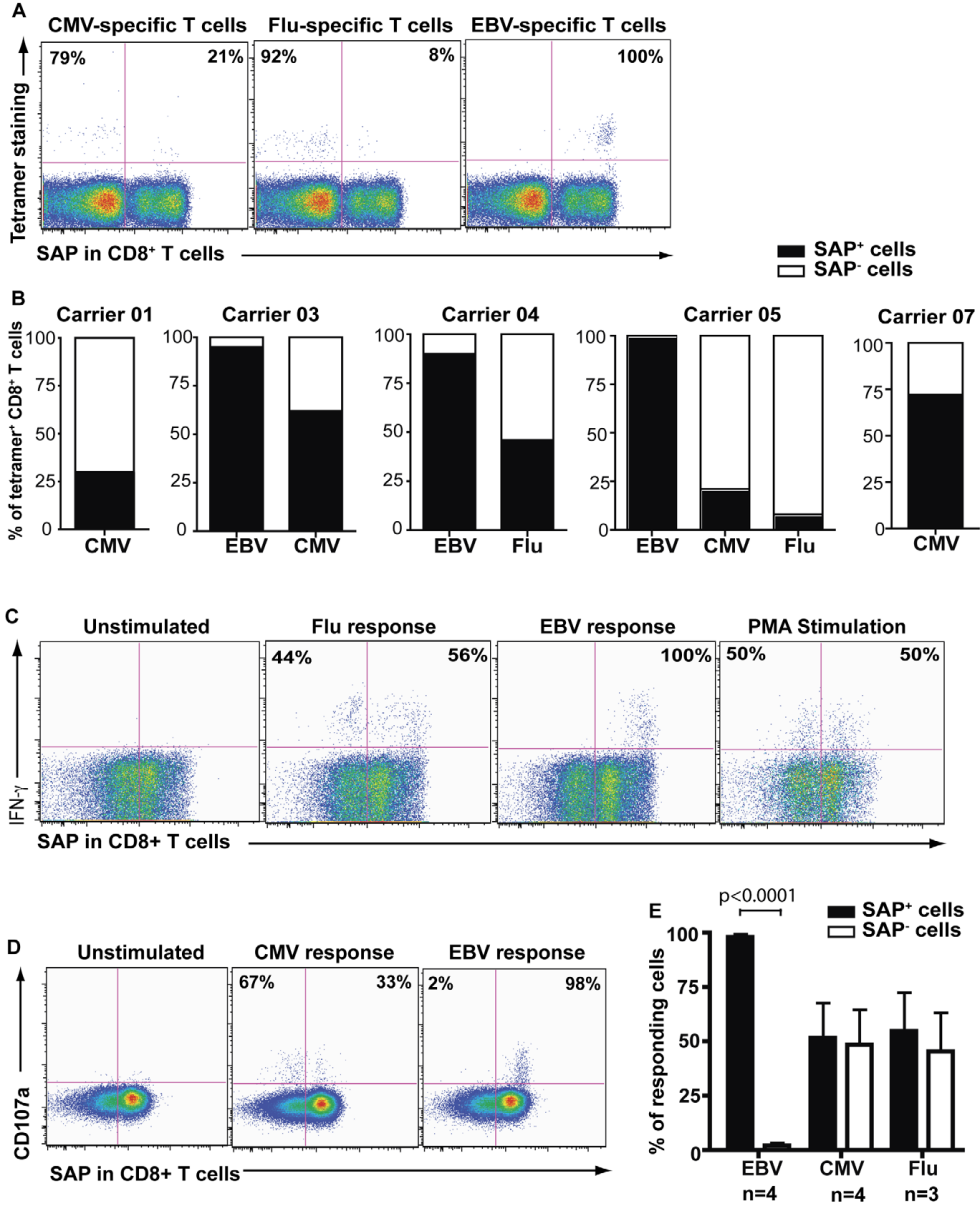
Selective recruitment of SAP^+^ cells into the EBV-specific memory CD8^+^ T cell compartment. (A, B) XLP carrier PBMC were labelled with specific MHC class I/peptide complexes together with anti-CD8 mAb; the cells were then fixed/permeabilised and incubated with anti-SAP mAb. The proportion of SAP^+^ and SAP^−^ cells that were specific for the different viruses was then determined. Dot plots in (A) depict SAP expression in tetramer^+^ cells from a carrier with detectable populations of CMV-, Flu-, and EBV-specific CD8^+^ T cells. The graphs in (B) depict proportions of SAP^+^ and SAP^−^ cells amongst EBV, CMV, or Flu-specific CD8^+^ T cells from five different XLP carriers. (C–E) PBMCs from XLP carriers were either unstimulated or stimulated with EBV, CMV, or Flu peptides, or with PMA/ionomycin. Expression of (C) IFN-γ or (D) CD107a by SAP^−^ and SAP^+^ CD8^+^ T cells was determined after 4–6 h. The values represent the proportion of responding cells that were SAP^−^ or SAP^+^. (E) Summary of data obtained from analysis of CD8^+^ T cells from different carriers to determine secretion of IFN-γ or degranulation (i.e., CD107a expression) by SAP^+^ and SAP^−^ cells in response to EBV, CMV, and Flu peptides. “*n*” represents the number of carriers studied for each viral response.

To determine the contribution of SAP^+^ and SAP^−^ CD8^+^ T cells to antiviral immunity, we analysed SAP expression in populations of memory CD8^+^ T cells that were specific for EBV, CMV, and influenza (Flu), as detected by soluble peptide:MHC class I complexes (i.e., tetramers). Five of the XLP carriers had MHC class I types that allowed epitope-specific cells to be visualised by this approach. The frequency of CMV and Flu-specific CD8^+^ T cells within the SAP^+^ population (CMV: range 21%–72%; mean ± sem: 46.3%±12.3%, *n* = 4; Flu: 8% and 46%; mean: 27.0%±19%) was not significantly different from that within the SAP^−^ population (CMV: 55.7%±12.3%, *n* = 4 [*p* = 0.78]; Flu: 73.0%±19%, *n* = 2) (Figure 3A,B). In stark contrast, almost all EBV-specific CD8^+^ T cells expressed SAP (95.0%±2.9% versus 5.0%±2.9% in SAP^−^ cells, *n* = 4; *p* = 0.004; Figure 3A,B). The same clear-cut distinction was seen when the functional response of virus-specific CD8^+^ T cells to various antigenic peptide challenges was assessed in vitro. Following stimulation of PBMCs from XLP carriers with CMV or Flu Ags, both SAP^+^ and SAP^−^ cells produced IFN-γ (Figure 3C,E) and expressed surface CD107a (Figure 3D,E), an indicator of the ability of cells to degranulate [44],[45]. However, when PBMCs were stimulated with various EBV peptides, including those from both lytic and latent Ags, only SAP^+^ CD8^+^ T cells responded (Figure 3C–E). Consistent with the recognition of EBV tetramers, the differences in the responses of SAP^+^ and SAP^−^ CD8^+^ T cells to in vitro stimulation with EBV peptides were highly significant (*p* = 0.0001; Figure 3E). Taken together these data demonstrated that the CD8^+^ T cell response to EBV infection in healthy XLP carriers had been preferentially recruited from SAP^+^ T cells, whereas the CD8^+^ T cell response to other viruses showed no preference for SAP-expressing cells.

### Phenotypic Features of SAP^−^ and SAP^+^ Cells

One explanation for the disparate responses of SAP^−^ and SAP^+^ CD8^+^ T cells to EBV, but not to other viruses, may result from differential expression of co-stimulatory or inhibitory molecules in the absence of SAP. Thus, we determined the phenotype of SAP^−^ and SAP^+^ cells with respect to expression of a suite of molecules known to regulate CD8^+^ T cell function. Expression of the co-stimulatory/activation/effector molecules CD27, CD28, CD38, OX40, ICOS, perforin, and granzyme B did not differ between SAP^−^ and SAP^+^ CD8^+^ T cells, irrespective of whether the cells were of a naïve or memory phenotype. Similarly molecules known to inhibit lymphocyte function—PD-1, BTLA—were comparably expressed on SAP^−^ and SAP^+^ naïve and memory CD8^+^ T cells (unpublished data). We also analysed the TCR repertoire of SAP^−^ and SAP^+^ cells by determining expression of distinct TCR Vβ chains by flow cytometry to deduce whether the TCR usage was significantly different between these cells. Although this approach may not be sufficiently sensitive to detect restricted diversity, the TCR repertories of SAP^−^ and SAP^+^ cells appeared to be generally similar (Table 1). The few biased TCR Vβ chains used in two carriers (#1, #3; Table 1) probably reflects the responses of different subsets of effector/memory cells to different viruses and their unique antigenic epitopes. Thus, lack of SAP expression does not appear to alter thymic selection of CD8^+^ T cells, or their ability to acquire expression of receptors involved in regulating lymphocyte function. Consequently, it is unlikely that perturbed selection or activation of SAP^−^ CD8^+^ T cells through co-stimulatory and regulatory receptors underlies their poor responsiveness to stimulation with EBV. Rather, this is likely a direct effect of SAP deficiency.

**Table 1:** TCR Vβ expression by SAP^−^ and SAP^+^ CD8^+^ T cells in XLP carriers.

TCR Vβ Chain	% CD8^+^ T Cells Expressing TCR Vβ Chains					
	XLP Carrier 1		XLP Carrier 3		XLP Carrier 4	
	SAP^−^	SAP^+^	SAP^−^	SAP^+^	SAP^−^	SAP^+^
**1**	4.0	1.45	3.35	3.68	4.77	5.45
**2**	3.32	0.85	2.33	3.71	8.3	5.97
**3**	0.62	0.47	0.55	0.39	0.85	1.28
**4**	1.61	0.27	1.53	1.71	3.35	1.8
**5.1**	1.9	5.95	2.6	17.1	4.2	2.9
**5.2**	1.16	0.49	1.1	0.52	1.68	2.8
**5.3**	1.02	0.27	4.4	1.93	4.64	6.37
**7.1**	12.6	0.42	1.8	3.08	5.37	3.85
**7.2**	1.25	2.62	1.62	3.2	1.98	3.96
**8**	3.06	7.18	1.35	4.14	4.1	7.22
**9**	0.54	0.22	0.22	1.61	0.71	1.54
**11**	4.80	34.10	19.8	6.3	4.93	4.74
**12**	1.53	0.5	1.04	0.8	0.98	0.9
**13.1**	4.4	9.81	4.7	2.5	4.37	4.27
**13.2**	1.46	0.5	1.37	2.1	1.48	1.31
**13.6**	0.88	0.27	2.9	0.12	1.2	1.14
**14**	0.7	0.37	9.85	1.0	0.84	0.4
**16**	1.50	0.59	0.86	1.06	2.3	2.44
**17**	3.67	3.35	3.9	2.25	7.36	5.78
**18**	0.79	0.35	0.94	1.5	1.1	0.94
**20**	0.39	0.34	0.34	0.61	1.7	0.9
**21.3**	1.03	0.52	1.21	4.5	0.8	1.0
**22**	2.90	0.9	2.3	1.14	4.2	3.0
**23**	1.81	0.35	3.35	1.65	3.7	2.58

### SAP Is Required for CD8^+^ T Cell-Mediated Cytotoxicity of Ag-Presenting B Cells

**Figure 4:**
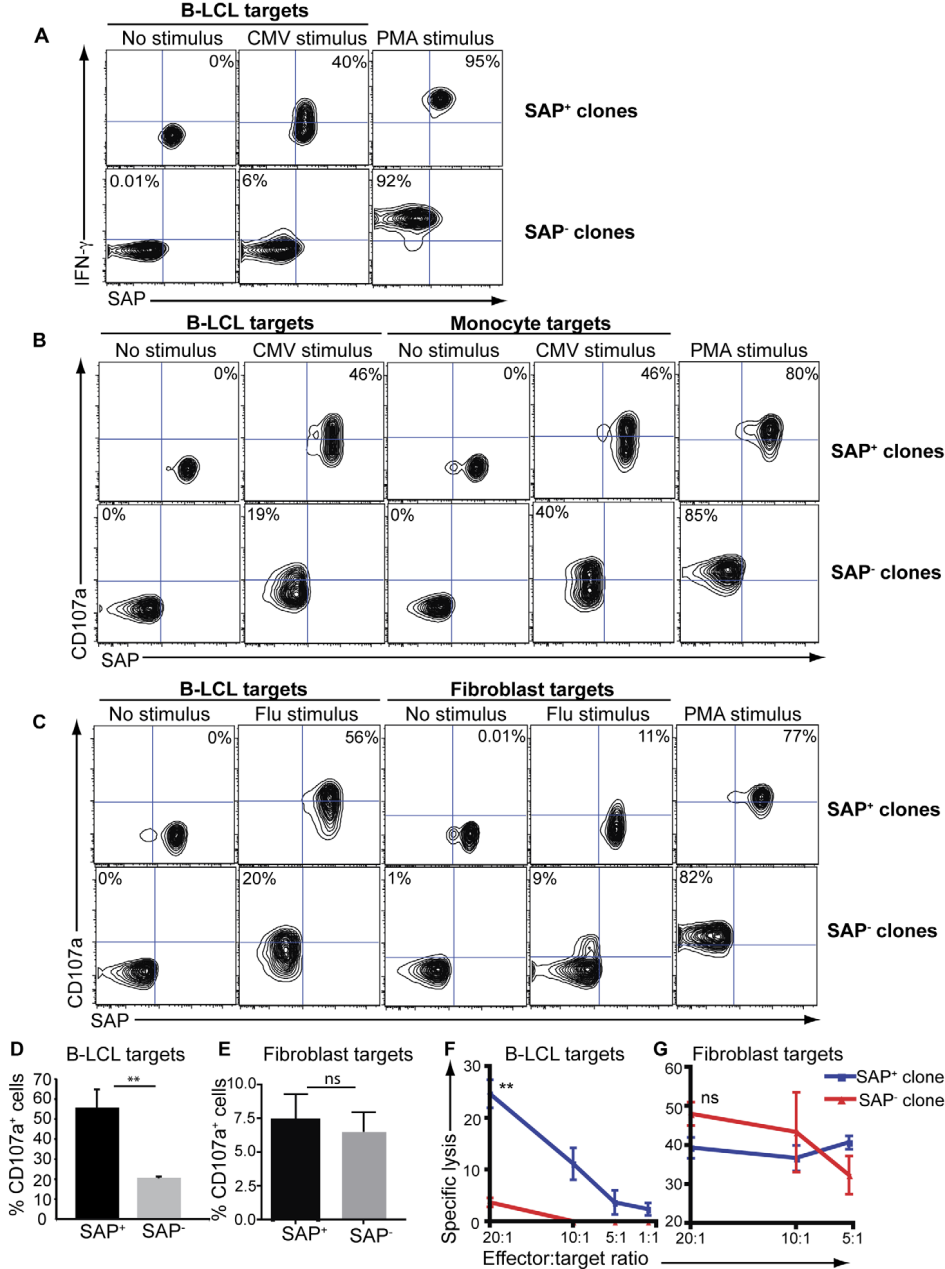
SAP deficient CD8^+^ T cells fail to respond to B cell targets. SAP^+^ and SAP^−^ CD8^+^ T cell clones specific for (A, B) CMV or (C–E) Flu isolated from unrelated XLP carriers were cultured with (A) autologous B-LCLs, (B) B-LCLs or HLA-matched monocytes, or (C–E) B-LCLs or HLA-matched fibroblasts that had been pulsed with either an irrelevant or cognate peptide for 4–6 h. Stimulation with PMA/Ionomycin was used as a positive control. Expression of IFN-γ (A) or CD107a (B–E) was then determined. The graphs in (D) and (E) represent the percentage of Flu-specific SAP^+^ or SAP^−^ cells induced to express CD107a^+^ following stimulation with peptide-pulsed B-LCLs (D) or fibroblasts (E). The values represent the mean ± sem of experiments using three different Flu-specific SAP^+^ or SAP^−^ clones. (F, G) SAP^+^ and SAP^−^ Flu-specific CD8^+^ T cell clones were cultured with ^51^Cr-labelled B-LCLs (F) or fibroblasts (G) pulsed with their cognate peptide for 4–6 h. Cytotoxicity was determined by standard chromium-release assay. The results are representative of two experiments performed using different clonal pairs of SAP^−^ and SAP^+^ cells. Data presented in Figure S2 for responses to CMV-pulsed B-LCLs and monocytes were obtained from experiments using different pairs of SAP^−^ and SAP^+^ clones. ** *p*<0.05.

The selective dependence of EBV-specific CD8^+^ T-cell-mediated immunity on SAP raised the question of which T-cell extrinsic mechanisms might explain the differences between the responses to EBV versus CMV and Flu. Since Ag presentation was a logical place to start, we developed an approach that would allow us to analyse the ability of SAP^−^ T cells to respond to distinct types of APCs. Thus, multiple SAP^−^ and SAP^+^ clonal pairs were established from different XLP carriers (Figure S1) and then tested for their ability to recognise cognate peptides presented on different APC targets, namely autologous EBV-transformed lymphoblastoid cell lines (B-LCLs), or HLA class I-matched monocytes or fibroblasts. SAP^+^ CD8^+^ T cell clones responded to their specific peptide regardless of the nature of the APC, as evidenced by enhanced IFN- γ production (Figure 4A, upper panels), acquisition of expression of CD107a (Figure 4B–E, Figure S2A upper panel) and lysis of Ag-presenting target cells (Figure 4F,G). In contrast, SAP^−^ CD8^+^ T cell clones responded poorly upon stimulation with peptide-pulsed B-LCLs compared to SAP^+^ clones, irrespective of whether the clones were specific for CMV (Figure 4A,B, Figure S2A lower panels) or Flu (Figure 4C lower panel, Figure 4D,F). Importantly the defective responses of SAP^−^ clones to specific Ag presented on B-LCLs did not reflect a generalised activation defect because these cells responded as well as SAP^+^ cells following PMA/ionomycin stimulation (Figure 4A–C, Figure S2A). Strikingly, the impairment was restricted to Ag presented in a B cell context. Thus, the same SAP^−^ CMV-specific or Flu-specific clones responded as well as their SAP^+^ counterparts to peptides presented on HLA-matched monocytes (Figure 4B, Figure S2), or fibroblasts (Figure 4C,E,G).

We extended these studies by assessing induction of CD107a expression by SAP^−^ and SAP^+^ CD8^+^ T cells within a CMV-specific T cell line in response to presentation of specific Ag by in vitro–derived dendritic cells (DCs) compared to B-LCLs. Although the frequency of total CD8^+^ T cells responding to CMV peptides was similar irrespective of whether B-LCLs or DCs were the APC (∼5%–6%), the SAP^+^ CD8^+^ T cells predominated the response when CMV-derived peptides were presented by B-LCLs (>90% of responding cells; Figure S2B). In contrast, both SAP^−^ and SAP^+^ CD8^+^ T cells responded to Ag-presenting DCs (35% and 65% of responding cells, respectively; Figure S2B). These findings are entirely consistent with the data for Ag-specific paired SAP^−^ and SAP^+^ clones (Figure 4, Figure S2A), and together provide compelling evidence for an important role for SAP in mediating CD8^+^ T cell recognition of B cell targets.

It would be ideal to also demonstrate that EBV-specific SAP-deficient CD8^+^ T cells are unable to respond to Ag endogenously presented by B cells. This could not be investigated using XLP carriers due to the extreme paucity of EBV-specific cells within the SAP^−^ subset of CD8^+^ T cells in these individuals (see Figure 3). To address this, we generated EBV-specific CD8^+^ T cell lines from an XLP patient with a well-characterised loss-of-expression mutation in *SH2D1A* ([F87S], XLP#3 in [46]). This was achieved by repeatedly expanding their purified CD8^+^ T cells on autologous EBV-transformed B-LCLs, as performed previously for other SAP-deficient patients [19]. As expected, EBV-specific CD8^+^ T cells from normal donors efficiently lysed autologous B-LCL target cells. In contrast, there was a profound defect in the ability of XLP CD8^+^ T cells to lyse autologous B-LCLs (Figure S2C, panel [i]). For these experiments, the donor and XLP patient were HLA matched. This allowed assessment of the ability of EBV-specific CD8^+^ T cells to lyse B-LCL derived from a SAP-sufficient donor or SAP-deficient XLP patient, and thereby to determine whether the cytotoxic defect of XLP CD8^+^ T cells resulted from impaired presentation of EBV Ag by SAP-deficient B-LCL. When this experiment was performed, XLP CD8^+^ T cells proved to be equally defective in killing allogeneic B-LCLs, which contrasted the behaviour of EBV-specific CD8^+^ T cell lines from normal donors (Figure S2C panel [ii]). Importantly, the inability of XLP CD8^+^ T cells to lyse B-LCL target cells did not appear to result from altered expression of lytic effector molecules since acquisition of perforin and granzyme B by XLP CD8^+^ T cells was comparable to that of normal CD8^+^ T cells (Figure S2C panel [iii]). This is consistent with the reduced cytotoxicity of SAP-deficient cells resulting from impaired recognition of B-LCL targets, which subsequently compromises immune synapse formation between effector and target cells, and polarisation of lytic mediators [19],[47].

### SAP^+^ and SAP^−^ T Cells Display Comparable Expression of the SLAM Family of Receptors, Yet Their Ligands Are Differentially Expressed by Distinct Types of APCs

**Figure 5:**
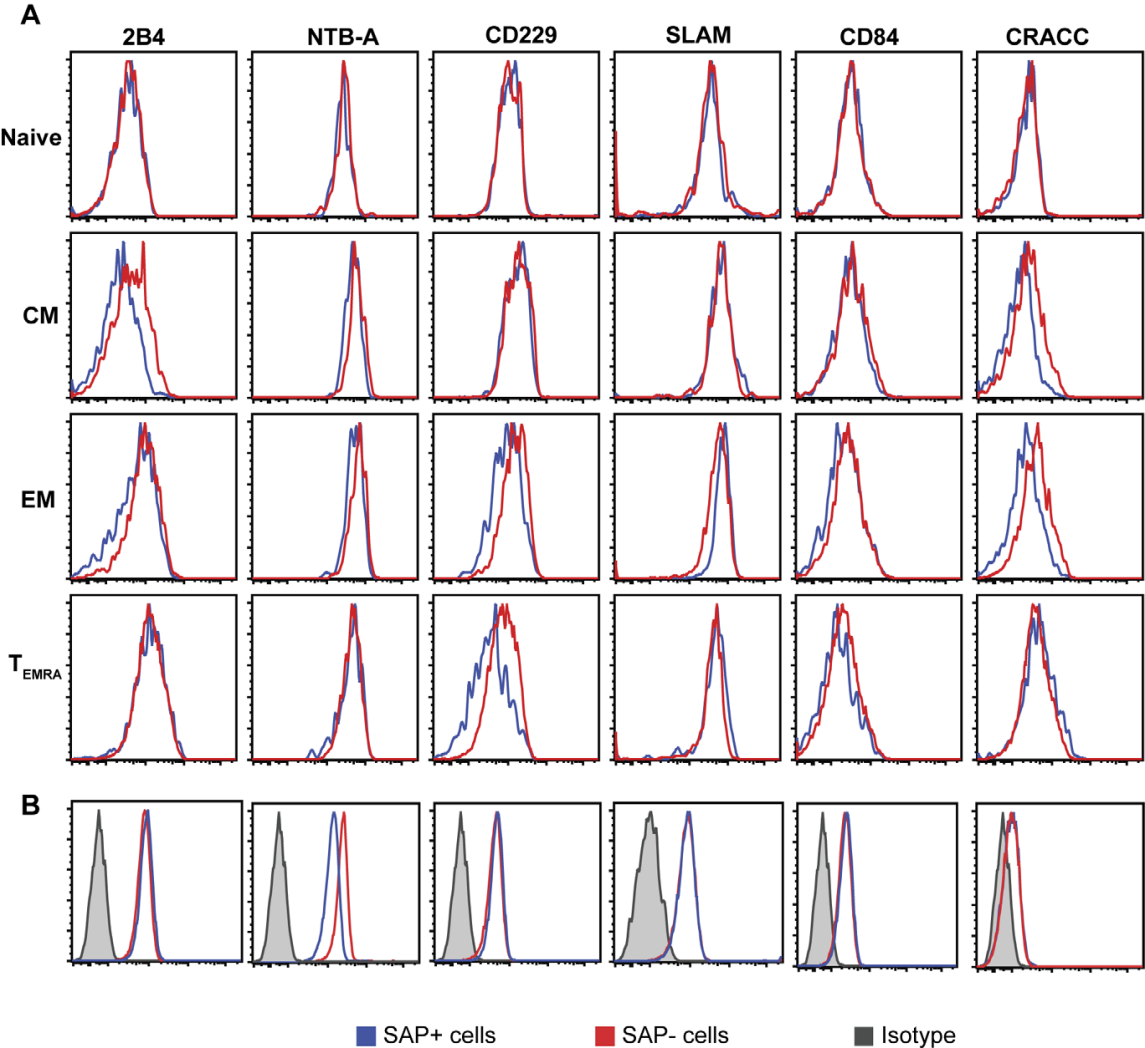
Expression of SLAM family receptors on CD8^+^ T cell subsets in XLP carriers. PBMCs from XLP carriers were stained with mAb specific for CD8, CD45RA, and CCR7 and either 2B4, NTB-A, CD229, SLAM, CD84, or CRACC; expression of SAP was then detected following fixation and permeabilisation. Expression of each of the SLAM family members on SAP^−^ and SAP^+^ naïve, central memory, effector memory, and T_EMRA_ CD8^+^ T cells was determined by gating on CD45RA^+^CCR7^+^, CD45RA^−^CCR7^+^, CD45RA^−^CCR7^−^, and CD45RA^+^CCR7^−^ cells, respectively. The histograms in (A) are derived from analysis of one carrier. Data for all carriers are presented in Figure S3. (B) Representative histogram plots of SLAM family receptor expression on SAP^+^ and SAP^−^ CD8^+^ T cell clones.

To begin to elucidate the mechanism underlying compromised SAP^−^ CD8^+^ T cell recognition of peptide-pulsed B cell targets and explore ways in which function might be restored, we examined the expression of SAP-associating receptors on subsets of SAP^−^ and SAP^+^ T cells. SAP associates with the cytoplasmic domains of SLAM, 2B4, CD84, NTB-A, CD229, and possibly CRACC [4],[7]. When expression of these molecules was assessed on lymphocytes from XLP carriers, we found no significant differences in their expression on SAP^−^ and SAP^+^ CD8^+^ T cells within the naïve and T_EMRA_ subsets (*p*>0.05; Figure 5A; Figure S3). Most of these molecules were also expressed comparably on SAP^−^ and SAP^+^ central memory and effector memory CD8^+^ T cells. However, there were significant differences in the expression levels of 2B4 and NTB-A on SAP^−^ and SAP^+^ central memory CD8^+^ T cells, and of 2B4 and CRACC on SAP^−^ and SAP^+^ effector memory CD8^+^ T cells, with them being lower on SAP^−^, relative to SAP^+^, cells. While these differences were statistically significant, the net differences in expression were <2-fold. Thus, it is unknown whether this would translate to a biological effect; furthermore, it is important to highlight that CRACC has been reported to function independently of SAP, at least in the context of human NK cells [48]. Thus, the lower level of CRACC on SAP^−^ cells will be inconsequential at least with respect to SLAM-receptor/SAP-dependent signalling and lymphocyte activation. These data generally imply that, at the cell surface, SAP^−^ and SAP^+^ CD8^+^ T cells are similarly capable of interacting with relevant ligands of the SLAM family.

**Figure 6:**
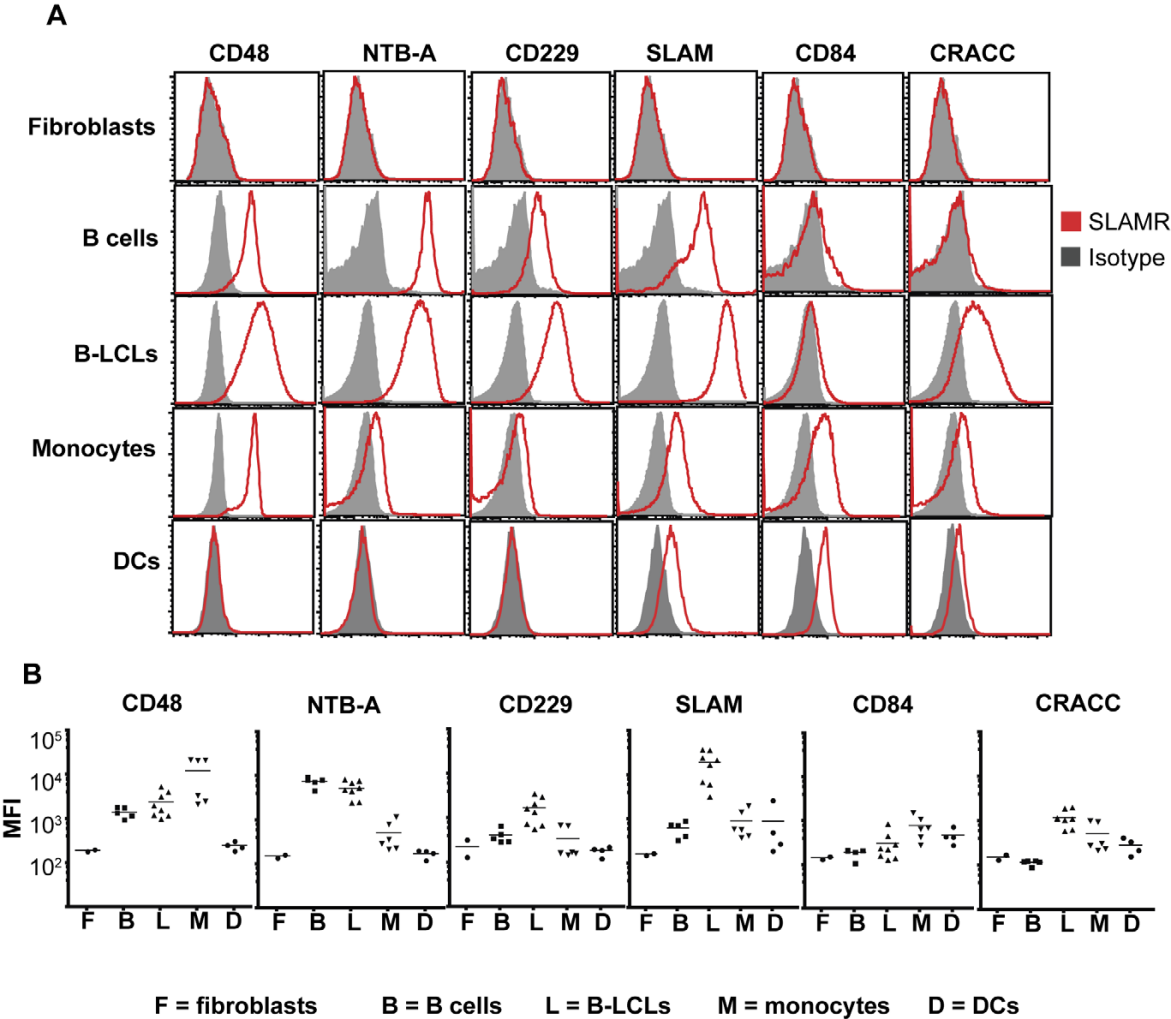
SLAM family receptor ligands are differentially expressed by distinct types of APCs. PBMCs from healthy controls (*n* = 6), B-LCLs from healthy controls and XLP carriers (*n* = 8), monocyte-derived DCs (*n* = 4), and human fibroblasts (*n* = 2) were stained with mAb specific for SLAM family receptors CD48, NTB-A, CD229, SLAM, CD84, or CRACC. Monocytes and B cells in the PBMCs were identified by expression of CD14 and CD20, respectively. DCs were identified by expression of CD1a, CD11c, and MHC class II. (A) Histograms of the expression of ligands of the SLAM family on human fibroblasts, resting primary B cells, B-LCLs, monocytes, and in vitro–derived DCs. (B) The mean fluorescence intensity of the expression of the different molecules on different APCs (F, fibroblasts; B, resting primary B cells; L, B-LCLs; M, monocytes; DC, dendritic cells).

The next step was to examine expression of ligands of the SLAM family receptors on different APCs because expression of these molecules on APCs could also influence the outcome of CD8^+^ T cell-mediated recognition of target cells. While 2B4 interacts with CD48, the other SLAM family receptors are self-ligands [4],[7]. In contrast to SAP^+^ and SAP^−^ CD8^+^ T cells, there were substantial differences in expression of SLAM family ligands by B-cell and non-B-cell APCs. NTB-A expression was highest on B cells and B-LCLs, while CD48 was highest on monocytes and B-LCLs (Figure 6A,B). B-LCLs also expressed higher levels of CD229, CRACC, and SLAM than resting B cells and monocytes (Figure 6A,B). Interestingly, NTB-A, CD48, and CD229 were all absent from in vitro–derived DCs; however, DCs did express CRACC, SLAM, and CD84 (Figure 6A,B). The relative levels of these molecules on DCs were similar to monocytes, with CRACC and SLAM being less, and CD84 being greater, than on B-LCLs (Figure 6A,B). Unlike APCs of hematopoietic origin, fibroblasts did not express any SLAM family ligands (Figure 6A,B). Thus, APCs exhibit substantial differences in their pattern of expression of SLAM family ligands.

### NTB-A and 2B4 Regulate CD8^+^ T Cells by Inhibiting Their Effector Function in the Absence of SAP

The above findings implied that engagement of distinct arrays of co-stimulatory receptors on SAP^−^ and SAP^+^ CD8^+^ T cells by ligands expressed on different APCs would modulate the acquisition of effector function of the responding CD8^+^ T cells. This would be consistent with the ability of SLAM family receptors to switch their function from activating or inhibitory depending on the presence of SAP [22],[24],[32]. We therefore explored the possibility that defined interactions between specific SLAM receptors on SAP^+^ or SAP^−^ CD8^+^ T cells and their ligands on APCs differentially regulated cytotoxicity.

**Figure 7:**
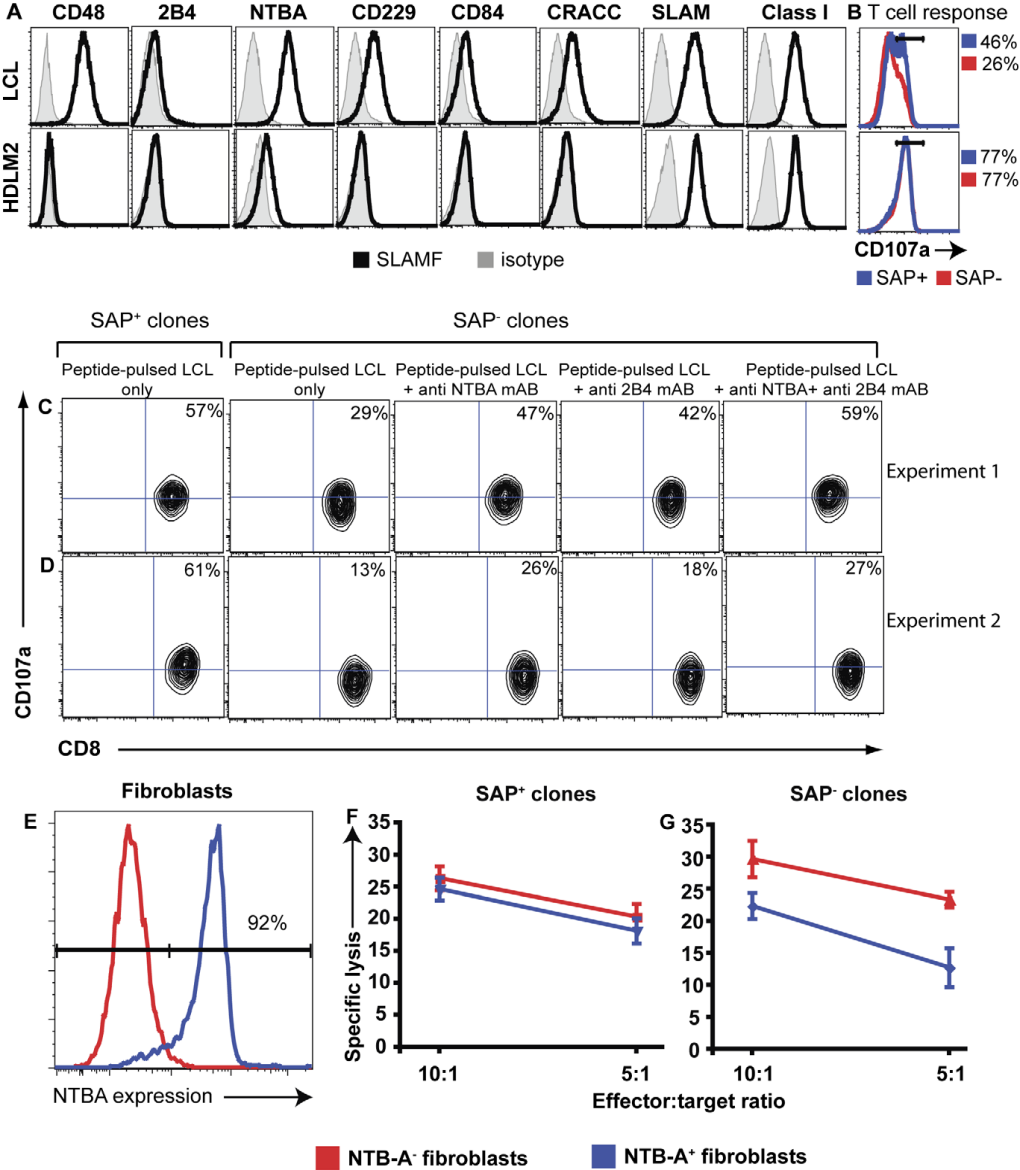
SLAM- family receptors inhibit the function of Ag-specific SAP^−^ CD8^+^ T cells. (A) Expression of SLAM receptors and MHC class I on B-LCL and the Hodgkin's lymphoma cell line HDLM2 were determined. (B) The ability of CD8^+^ T cells to be activated by B-LCL and HDLM2 cells was assessed by incubating CMV-specific SAP^−^ (red histogram) and SAP^+^ (blue histogram) CD8^+^ T cells with peptide-pulsed target cells. The values represent the percentage of CD107a-expressing SAP^−^ and SAP^+^ cells detected after 4–6 h incubation with the different target cells. (C, D) SAP^+^ and SAP^−^ CD8^+^ T cell clones specific for CMV were cultured with peptide-pulsed autologous B-LCLs in the presence or absence of specific mAb to NTBA alone, 2B4 alone, or in combination. Expression of CD107a by SAP^−^ and SAP^+^ CD8^+^ T cells was determined after 4–6 h. The values represent the proportion of responding cells. The data presented in (C) and (D) represent independent experiments performed using different pairs of CMV-specific CD8^+^ T cell clones. (E) Expression of NTB-A on parental fibroblasts (red histogram) or those transfected to express NTB-A (blue histogram). (F, G) SAP^+^ (F) and SAP^−^ (G) CMV-specific CD8^+^ T cells clones were cultured with ^51^Cr-labelled parental (red) or NTB-A-expressing (blue) fibroblast target cells. Cytotoxicity was determined after 4 h and is expressed as percentage of Target cell lysis. Each value is the mean ± sem of triplicate samples and is representative of experiments performed using three different pairs of SAP^−^ and SAP^+^ CMV-specific CD8^+^ T cell clones.

We first examined the ability of SAP^+^ and SAP^−^ CD8^+^ T cells to respond to the Hodgkin's lymphoma cell line HDLM2. This line was chosen as a target cell because (a) it lacked expression of all SLAM family ligands with the exception of SLAM/CD150 itself (Figure 7A), (b) SLAM has been reported to enhance the cytotoxicity of human CD8^+^ T cells [49], and (c) SLAM was expressed at the highest levels on B cells relative to other APCs (Figure 6), revealing it as a candidate molecule to regulate CD8^+^ T cell function. Thus, if expression of SLAM on B cells, but not fibroblasts, controls the effector function of CD8^+^ T cells, then it would be predicted that SAP^−^ CD8^+^ T cells would exhibit reduced cytotoxicity against HDLM2 cells than their SAP^+^ counterparts. When this was tested experimentally by pulsing either autologous B-LCLs or MHC class I–matched HDLM2 cells with CMV peptides and assessing the response of CMV-specific CD8^+^ T cells, both SAP^−^ and SAP^+^ cells were equally capable of responding to HDLM2, as evidenced by acquisition of CD107a expression by a comparable proportion of cells (Figure 7B, lower panel), but not to B-LCLs, as expected (Figure 7B, upper panel). This dichotomy in recognising and responding to B-LCLs versus HDLM2 was not due to differences in expression of MHC class I by the target APCs (Figure 7A). This finding suggested that SLAM was unlikely to be the predominant receptor mediating the effector function of CD8^+^ T cells in the absence of SAP.

This led us to focus on NTB-A and 2B4 because their ligands (i.e., NTB-A, CD48) are highly expressed on B cells (Figure 6; [22],[50]) and they can deliver activating and inhibitory signals in the presence and absence, respectively, of SAP to human NK and CD8^+^ T cells [22],[24],[26],[27],[32]. Although CRACC was also more highly expressed on human B-LCLs than on monocytes (Figure 6), its role in regulating CD8^+^ T cell function was not explored because it functions independently of SAP [48],[51].

When interactions between NTB-A/NTB-A and/or 2B4/CD48 were blocked with specific mAbs [22],[52]–[54], activation of SAP^+^ CD8^+^ T cells by B cell targets was not significantly affected (%CD107a^+^ cells—no mAb: 51.3%±3.8%; + anti-NTB-A mAb: 56%±6.5%; + anti-2B4 mAb: 55.7%±5.6%; + anti-NTB-A/2B4 mAbs: 55.7%±7.3%; *n* = 4, *p* = 0.48 [27],[32]). By contrast, blocking interactions between NTB-A/NTB-A or 2B4/CD48 substantially improved the effector function of SAP^−^ CD8^+^ T cells compared to when these cells were examined in the absence of added mAbs (Figure 7C,D). Importantly, combined blockade of both pathways could restore effector function of SAP^−^ T cells to a level comparable to SAP^+^ clones (Figure 7C). These observations suggest that signalling through NTB-A and 2B4 impedes the effector function of SAP-deficient, but not SAP-sufficient, CD8^+^ T cell in response to Ag-presenting B cell targets.

To provide additional data that homotypic NTB-A interactions can suppress the function of SAP-deficient CD8^+^ T cells, we transfected fibroblasts to express NTB-A (Figure 7E) and compared the ability of SAP^+^ and SAP^−^ clones to lyse the parental (i.e., NTB-A^−^) or transduced NTB-A^+^ cells in a ^51^Cr release assay. Consistent with the data presented in Figure 4, there was no difference in lysis of either parental fibroblasts by SAP^+^ and SAP^−^ CD8^+^ T cell clones (compare Figure 7F and G; red lines), or lysis of NTB-A^−^ and NTB-A^+^ fibroblasts by SAP^+^ CD8^+^ T cells clones (Figure 7F). However, the cytotoxic activity of the same SAP^−^ CD8^+^ T cell clone was significantly reduced when NTB-A was ectopically expressed on fibroblasts (Figure 7G, *p*<0.05). Thus, these data provide evidence that in the absence of SAP, SLAM family receptors acquire inhibitory function which compromises the ability of CD8^+^ T cells to be activated by Ag-presenting B cells.

## Discussion

Primary immune deficiencies are characterised by increased susceptibility to infection by a range of pathogens [10]. The molecular mechanism underlying this heightened vulnerability is often explained by the nature of the genetic defect responsible for a particular immune deficient condition. Thus, a lack of B cells in X-linked agammaglobulinemia (XLA) a lack of T and NK cells in X-linked several-combined immunodeficiency (X-SCID) and impaired B-cell responses in X-linked hyper-IgM syndrome due to mutations in *BTK*, *IL2RG*, and *CD40LG*, respectively, predispose affected individuals to severe, recurrent, and often life-threatening infections [10],[55]. In contrast to these conditions, the explanation for why loss-of-function mutations in *SH2D1A*, resulting in SAP-deficiency, render XLP patients exquisitely sensitive to infection with EBV, but not other viruses, is enigmatic. Indeed, while previous studies that examined lymphocytes from XLP patients or *Sap*-deficient mice have clearly shed light on the role of SAP in different immune cells and allowed us to understand the complex nature of some of the clinical manifestations of XLP [4],[7], the question of why XLP patients are uniquely susceptible to EBV infection remains unanswered. Efforts to address this have also been hampered by the absence of appropriate animal models due to the specificity of EBV infection for humans. For these reasons, we developed a novel approach to answer this basic question relating to XLP.

Female carriers of several X-linked diseases, such as X-SCID, XLA, and Wiskott-Aldrich syndrome, display skewed X-chromosome inactivation with preferential expression of the wild-type (WT) allele in some lymphocyte lineages [56]–[58]. This occurs because expression of the WT allele in specific hematopoietic cells confers a survival advantage over cells expressing the mutant allele, which therefore fail to develop in the female carriers. In contrast to these X-linked diseases, normal numbers of T and NK cells are detected in XLP patients [11],[16], and lymphocytes from female carriers of XLP exhibit random inactivation of the X-chromosome [11]. These observations demonstrate that SAP is not required for lymphocyte development (with the exception of NKT cells [11]; Figures 1, 2). Consequently, female carriers of XLP represent an ideal model to assess the role of SAP in CD8^+^ T cell-mediated anti-viral immune responses because both SAP^+^ and SAP^−^ cells with the same genetic background are generated at similar frequencies (Figure 2). This is essentially the human equivalent of a mixed bone marrow chimera in mice, and therefore eliminates any variability that may arise from comparisons of SAP-deficient CD8^+^ T cells from XLP patients with SAP-sufficient cells from unrelated normal donors, as has been performed in earlier studies [19],[20],[32]. Another feature of female XLP carriers is that they have an intact immune system and are not susceptible to any known infections [38],[39]. Thus, any secondary defects in the function of CD8^+^ T cells from XLP patients due to a lack of NKT cells or impaired NK cell function—which can all contribute to fine-tuning CD8^+^ T cell responses [33]–[36]—are circumvented by studying XLP carriers. These attributes of XLP carriers allowed us to perform a detailed analysis of the responses of SAP^−^ and SAP^+^ CD8^+^ T cells from the one individual to not only EBV but other common viruses including CMV and Flu in the setting of a normal host immune response.

Previous studies using tetramers have demonstrated that EBV-specific CD8^+^ T cells could be detected in XLP patients (*n* = 2; [59]). These cells, however, exhibit poor in vitro responses to EBV Ags [19],[32]. Our phenotypic and functional analysis of Ag-specific CD8^+^ T cells from XLP carriers demonstrated that CMV or Flu-specific CD8^+^ T cells are distributed within both SAP^+^ and SAP^−^ memory populations, however there was a dramatic, and highly significant, skewing of EBV-specific CD8^+^ T cells such that >95% of these cells were detected within the SAP^+^ compartment (Figure 3). By using peptides derived from both lytic and latent EBV Ag, we established that the exclusive SAP^+^ effector CD8^+^ T cells generated following EBV infection were not restricted to a single dominant antigenic epitope (Figure 3). This demonstrates that there is a selective advantage for SAP^+^ CD8^+^ T cells in anti-EBV immunity, but not in either anti-CMV or anti-Flu immunity. Thus, although SAP^−^ cells are abundant within the pool of naïve CD8^+^ T cells, the SAP^+^ cells expressing a TCR with specificity for EBV vigorously outcompete their SAP^−^ counterparts and subsequently become the predominant cell type that expands and is maintained following exposure to EBV. Thus, our studies reveal a strong requirement for SAP expression not only in mediating the effector function of CD8^+^ T cells in response to EBV infection but also in the expansion and survival of these cells. These findings underscore the obligate requirement for SAP, and by extension SLAM family receptors, at multiple stages in CD8^+^ T cells in mediating protection against EBV infection. The ability to examine competition between WT and gene-deficient cells ex vivo is another powerful feature of the carrier model, and a human equivalent of the studies performed in mice using mixed bone marrow chimeras to determine the intrinsic responses of WT versus mutant cells in a competitive environment.

The mechanism underlying this fundamental requirement for SAP expression during the generation of EBV-specific CD8^+^ T cells was revealed by investigating the ability of SAP^−^ and SAP^+^ CD8^+^ T cells specific for the same CMV or Flu epitopes to respond to their cognate peptide when presented on B-cell or non-B-cell target APCs (monocytes, DCs, fibroblasts). The rationale for these experiments was 2-fold: first, one of the key differences between the three viruses studied here is the identity of the APC responsible for activating the CD8^+^ T cell response. CMV persists in immature myeloid cells and, on reactivation, is likely to be presented by infected monocytes/DCs [60], whereas influenza infects respiratory epithelial cells and can be cross-presented by DCs [61]. By contrast, EBV is a predominantly B-lymphotrophic virus and there is strong evidence to suggest that the CD8^+^ T cell response is driven by epitopes displayed on infected B cells themselves [37],[62]. Second, although the response of XLP CD8^+^ T cells to B cells is impaired, they can respond relatively normally to other types of target cells [19],[32]. Thus, it was possible that SAP-deficient CD8^+^ T cells failed to be activated when Ag was specifically presented by B cells. Indeed, SAP-deficient CD8^+^ T cell clones from XLP carriers were specifically defective in responding to their cognate epitopes when presented by B-cell, but not non-B-cell, targets irrespective of the viral origin of the specific Ag (Figure 4). Similarly, EBV-specific SAP-deficient CD8^+^ T cells expanded from XLP patients were severely compromised in their capacity to lyse B cells presenting endogenously processed EBV peptide Ags (Figure S2C). Our findings have several important implications. First, although EBV can presumably be presented by numerous non-B-cell types of APCs (e.g., tonsillar epitheilium, cross-primed DCs) [63],[64], and this may contribute to the initial generation of detectable EBV-specific CD8^+^ T cells in XLP patients [19],[59], the predominant APC involved in maintaining a robust anti-EBV CD8^+^ T cell–mediated immune response appears to be B cells. Second, the inability to control EBV infection in XLP is likely to result from a direct defect in CD8^+^ T cells. Defects in CD4^+^ T cells may contribute to impaired anti-EBV immunity in XLP because analysis of the CD4^+^ T cell compartment from XLP carriers revealed a predominant response by SAP^+^ CD4^+^ T cells to EBV lysate in vitro (Figure S4). Third, and most importantly, the exquisite sensitivity of XLP patients to EBV infection results from the ability of the virus to sequester itself in infected B cells which can only induce a cytotoxic T cell response in SAP-sufficient cells. In other words, the functional defect in SAP^−^ CD8^+^ T cells does not relate to a specific virus but rather to the nature of the target cell presenting viral epitopes.

The finding of a requirement for SAP in CD8^+^ T cell–mediated lysis of Ag-presenting B cells, but not monocytes, DCs, or fibroblasts, predicted that expression of ligands of the SLAM family would differ between these populations of APCs. This was confirmed by demonstrating that while fibroblasts lacked expression of all SLAM family ligands, B cells, monocytes, and DCs expressed differing levels of some of these ligands (Figure 6). Signalling downstream of SLAM family receptors is regulated by SAP via several mechanisms. SAP can deliver activation signals via Fyn-dependent or Fyn-independent processes [6]. Alternatively, SLAM family receptors can alter their function to become inhibitory receptors in the absence of SAP [5],[6]. This appears to be mediated by the recruitment and/or activation of inhibitory phosphatases [22],[24],[65],[66]. We therefore reasoned that engagement of SLAM receptors delivered either activating signals to SAP-expressing CD8^+^ T cells or inhibitory signals to SAP-deficient CD8^+^ T cells. Our finding that (1) impeding NTB-A/NTB-A and 2B4/CD48 interactions with blocking mAbs [22],[52],[54] could improve the function of SAP^−^ CD8^+^ T cells in the context of responses to Ag-presenting B cell targets and (2) ectopic expression of NTB-A on fibroblasts protected these cells from cytotoxicity induced by SAP-deficient Ag-specific CD8^+^ T cells favoured an inhibitory function for these receptors in the absence of SAP (Figure 7). This is reminiscent of early descriptions of inhibitory function of these receptors on SAP-deficient human NK cells [22],[24],[67],[68], and the recent demonstration of such a phenomenon for CD8^+^ T cell clones from XLP patients [32]. This conclusion is also consistent with the reported ability of NTB-A to associate with SHP-1 in the absence of SAP in human NK cells and T cells [22],[42], thereby suggesting a mechanism of how NTB-A exerts its inhibitory effect. Veillette and colleagues proposed that the SAP homolog EAT-2 mediates inhibitory signalling downstream of some SLAM family receptors in the absence of SAP [69]. Interestingly, EAT-2 associates with NTB-A in human lymphocytes [70], and *SH2D1B* (encoding EAT-2) was expressed at increased levels in memory CD8^+^ T cells from XLP patients compared to healthy donors (Figure S5). Thus, it is possible that in XLP heightened expression of EAT-2 mediates an alternative pathway downstream of NTB-A for inhibitory signalling in SAP-deficient CD8^+^ T cells following engagement of SLAM family receptors. Irrespective of these possibilities, it is clear that expression of SAP significantly alters the function of SLAM family receptors on human NK and CD8^+^ T cells such that these receptors inhibit cytotoxicity in the absence of SAP.

Previous studies established defects in SAP-deficient CD8^+^ T cells [19],[20],[32]. However, there have been major limitations to all of these inasmuch as they only examined responses of XLP CD8^+^ T cells to polyclonal (i.e., Ag non-specific) stimulation [19],[20], or only studied responses to EBV and not additional viruses [19],[32]. Thus, none of these earlier studies offered an explanation for the selective inability of XLP patients to respond to infection with EBV but not other viruses. We have now significantly extended these observations by providing mechanistic insight into the dysfunctional behaviour of SAP^−^ CD8^+^ T cells by (1) revealing that the defect in anti-EBV immunity in XLP reflects the nature of the APC, rather than EBV itself, (2) proving that NTB-A is inhibitory for the function of SAP-deficient CD8^+^ T cells, and (3) excluding a role for SLAM itself in regulating the function of human Ag-specific CD8^+^ T cells, a scenario proposed by a previous study [49].

Our findings that SAP-deficient CD8^+^ T cells respond poorly to EBV-infected B cells, but not to monocyte, DC, or fibroblast APCs, parallel those reported recently for CD4^+^ T cells from *Sap* ^−*/*−^ mice. In that system no difference was found in the quality of interactions between DCs and either SAP-deficient or SAP-sufficient CD4^+^ T cells [17]. However, SAP-deficient CD4^+^ T cells exhibited greatly reduced interactions with cognate B cells, resulting in impaired help for T-dependent B cell responses [17]. Interestingly, mouse Ly108 (i.e., human NTB-A) is involved in the formation of stable conjugates between normal CD4^+^ T cells and B cells, while interactions with DCs were predominantly mediated by integrins [71]. The absence of NTB-A and CD48 from DCs potentially explains why DC-mediated Ag-presentation to CD8^+^ T cells is unaffected by SAP deficiency. While SAP was required in murine CD4^+^ T cells for NTB-A-mediated interactions with B cells [71], it appears that SAP functions in human CD8^+^ T cells to prevent the delivery of inhibitory signals downstream of NTB-A that probably involve the recruitment and/or activation of phosphatases or EAT-2 [22],[42],[70]. This apparent disparate function of NTB-A on murine CD4^+^ and human CD8^+^ T cells may be explained by the pattern of expression of EAT-2, inasmuch as it is detected in human CD8^+^ T cells (Figure S5) [72], but not murine CD4^+^ T cells [69]. Despite these potential differences, an emerging theme is that loss of SAP in T cells leads to altered interactions with B cells, while interactions with other APCs remain intact. This specific defect not only explains the molecular pathogenesis of the unique susceptibility to EBV infection in XLP patients but potentially explains their high incidence of B-lymphomas. Interestingly, EBV is the only known human pathogen that selectively infects B cells, which results in expression of high levels of SLAM family ligands to facilitate the T-B cell cross-talk necessary for immunity. Thus, our studies have identified a unique pathological signalling pathway that may be targeted to treat patients with severe EBV infection. Furthermore, the innovative XLP carrier model has allowed us to unravel the mechanisms of disease in the absence of a relevant animal model. This system may also allow the study of other human diseases, for instance XIAP deficiency, which also predisposes to EBV infection [8],[73], where heterozygous gene expression from random X-chromosome inactivation could be exploited.

## Materials and Methods

### XLP Carriers and Patients

Blood samples were collected from seven different XLP carriers and an XLP patient. PBMC were isolated and either used fresh or cryopreserved in liquid nitrogen. Genomic DNA was sequenced to confirm the heterozygous state of the carriers. Primers used for amplification of the four exons of *SH2D1A* are: Exon 1 sense: CAA CAT CCT GTT GTT GGG G, Exon 1 antisense: CCA GGG AAT GAA ATC CCC; Exon 2 sense: GCA ATG ACA CCA TAT ACG, Exon 2 antisense: GAA CAA TTT TGG ATT GGA GC; Exon 3 sense: GTA AGC TCT TCT GGA ATG, Exon 3 antisense: CAT CTA CTT TCT CAC TGC; Exon 4 sense: CTG TGT TGT GTC ATT GTG, Exon 4 antisense: GCT TCC ATT ACA GGA CTA C. All participants gave written informed consent and the experiments were approved by the Human Research Ethic committees of the Sydney South West Area Health Service (Royal Prince Alfred and Concord Zones) and St. Vincent's Hospital.

### Flow Cytometric Analysis

PBMC, CD8 T cell clones, B-LCLs, and fibroblasts were stained with fluorochrome-conjugated mAbs specific for cell surface receptors. The following mAbs were used to identify different lymphocyte populations: anti-CD3, CD4, CD8 (T cells), CD56 (NK cells), CD20 (B cells), CD14 (monocytes), CD1a, CD11c (DC) (BD Biosciences), and TCR Vα24/Vβ11 (NKT cells) (Immunotech, France) mAbs. CCR7 (R&D Systems), CD45RA (BD Biosciences), and CD27 (BD Biosciences) were used to identify subsets of naïve and memory T and B cells. CD83 (eBioscience), CD86, MHC class II, and MHC class I mAbs (BD Biosciences) were used to phenotype LPS-matured DCs. Expression of the SLAM family of receptors and ligands was determined using mAbs against CD84 (BD Biosciences), CD229, NTBA, CRACC (R&D Systems), 2B4 (Beckman Coulter), CD48 (Immunotech, France), and SLAM/CD150 (eBiosciences). TCR Vβ repertoire analysis was performed according to the manufacturer's instructions (Beckman Coulter). For degranulation assays mAb against CD107a (BD Biosciences) was used as previously described [44],[45] and for intracellular cytokine stains anti-IFN-γ (BD Biosciences) mAb was used. Stained cells were analyzed on either FACSCanto I or II flow cytometers (BD Biosciences) and the data processed using FlowJo software (Treestar, Ashland, USA).

### MHC Class I Tetramers

MHC class I tetramers were prepared in-house, where the appropriate MHC class I heavy chain molecule was refolded with β2 microglobulin and the peptide and complexed with streptavidin-PE as described [74]. CMV epitopes used were the HLA-A*0201-restricted peptides NLVPMVATV from pp65 (UL83) protein, and VLEETSVML from IE-1 (UL122) protein; HLA-A*0101 restricted peptide, VTEHDTLLY from pp50 (UL44) protein. EBV epitopes used were HLA-A*0201-restricted GLCTLVAML from the lytic Ag BMLF-1, CLGGLLTMV from LMP2, HLA-B*4402-restricted peptides VEITPYKPTW from EBNA3B latent protein, and EENLLDFVRF from EBNA3C. The influenza A epitope was the HLA-A*0201-restricted peptide GILGFVFTL from matrix protein.

### Detection of SAP by Intracellular Staining

Cells were first stained for surface markers and then fixed with 2% paraformaldehyde, permeabilized with 0.5% saponin, and incubated with Alexa Fluor 647 (Invitrogen)-conjugated isotype control or anti-SAP mAb (Abnova, clone 1C9). Cells were washed and resuspended in PBS/1% FCS and analysed on a FACSCanto I or II flow cytometer (BD Biosciences).

### PBMC Stimulation

1–2×10^6^ PBMCs were stimulated with either an irrelevant peptide, specific MHC class I restricted synthetic peptide, or PMA/ionomycin as a positive control for 4–6 h in the presence of Brefeldin A (for IFN-γ production) or monensin (for CD107a expression). The capacity to respond to these peptides was tested by harvesting the cells and determining expression of IFN-γ or CD107a by SAP^+^ and SAP^−^ CD8^+^ T cells.

### Generation and Culture of Human Monocyte-Derived Dendritic Cells

DCs were generated from peripheral blood monocytes by culturing sort-purified CD14^+^ cells (5×10^5^/ml) in human lymphocyte media [15],[16] supplemented with 500 U/ml of IL-4 (provided by Dr. Rene de Waal Malefyt) and 50 ng/ml GMCSF (Peprotech). After 5 d, monocyte-derived DCs were harvested, washed, and cultured (5×10^5^/ml) in the presence of 1 µg/ml of LPS (Sigma) for a further 18 h. Monocyte-derived DCs were CD1a^+^ CD11c^+^ CD14^−^. Upon maturation with LPS, they upregulated expression of CD83, CD86, and MHC class I and MHC class II.

### Generation of Ag-Specific T Cell Clones and Lines

Virus-specific CD8^+^ T cell clones were established from PBMCs by sort-purifying tetramer positive cells and limiting dilution cloning as described [75]. Clones were established by seeding sort-purified tetramer^+^ CD8^+^ T cells at 0.3–3 cells/well into media containing 10^4^ autologous B-LCLs and 10^5^ feeder cells per well. CMV-specific clones were selected based on their recognition of the pp50 (UL44) epitope VTEHDTLLY (HLA-A1 restricted), while influenza-specific clones recognised the matrix protein epitope GILGFVFTL (HLA-A2 restricted). All clones were expanded and tested for specificity by staining with the appropriate tetramer and for SAP expression (see Figure S1). EBV-specific CD8^+^ T cell lines used in DC assays were generated by sort purifying tetramer-positive cells and expanding them in vitro on peptide-pulsed autologous B-LCLs and feeder cells. EBV-specific CD8^+^ T cell lines from XLP patients and normal donors were established by repeated stimulation of purified CD8^+^ T cells on autologous B-LCLs [19].

### T Cell Recognition Assay

The ability of CD8^+^ T cell clones to respond to various target cells was measured either by intracellular IFN-γ staining or by staining for CD107a. Autologous B-LCLs were used as B cell targets. HLA-matched monocytes were sort-purified from buffy coats on the basis of CD14 (Immunotech) expression and used as APCs. DCs were generated as described above. HLA-matched human fibroblasts used were JuSt (HLA-A1 & A2) and MeWo cells (HLA A2) (ATCC). All APCs were pulsed with appropriate peptides (1 µg/ml) and used to stimulate CD8^+^ T cell clones. Where cytotoxicity was measured, APCs were sensitised with cognate peptide at a concentration of 1 µg/ml while loading with ^51^Cr. After washing, T cells were incubated at different APC∶T cell ratios and incubated for 5 h in standard cytotoxicity assay [75]. In some experiments, blocking mAbs against NTB-A (MA127) [22] and 2B4 (C1.7 [52],[53]) were used to prevent NTB-A/NTB-A and 2B4/CD48 interactions, respectively. B-LCLs were incubated with the relevant mAb at a final concentration of 20 µg/ml for 30–45 min prior to mixing with CTL clones. Cultures were incubated for 4–6 h in the presence of blocking mAbs and mAb to CD107a. Cells were then appropriately stained and analysed by flow cytometry. Fibroblasts were transfected using Lipofectamine with the pcdef3 plasmid containing cDNA encoding human NTB-A. Positive cells were initially selected in the presence of G418 and then isolated by sorting NTB-A^+^ cells. NTB-A^+^ transfected and untransfected parental fibroblasts were then used as targets in ^51^Cr release assay as described above.

## Supporting Information

Figure S1 Generation of SAP^−^ and SAP^+^ virus-specific clones. Virus-specific cells were isolated from PBMCs of XLP carriers by sorting tetramer^+^ cells (A). Clones were then established by limiting dilution assay and positive clones were expanded. All clones were then examined for their expression of SAP by intracellular staining (B) and specificity by tetramer staining (C).
(TIF)

Figure S2 SAP deficient CD8^+^ T cells fail to respond to B cell targets. (A) Ag-specific SAP^+^ (upper panel) and SAP^−^ (lower panel) CD8^+^ T cell clones or (B) EBV-specific CD8^+^ T cell lines isolated from an XLP carrier were cultured with (A) autologous B-LCLs or HLA-matched monocytes or (B) autologous B-LCLs or HLA-matched DCs that had been pulsed with either an irrelevant or cognate peptide for 4–6 h. Stimulation with PMA/Ionomycin was used as a positive control. Expression of CD107a was then determined. These results are derived from different sets of clones as those presented in Figure 4. (C) EBV-specific CD8^+^ T cell lines were established from a healthy control or an XLP patient. The ability of these cells to lyse autologous (panel [i]) and allogeneic but HLA-matched (panel [ii]) B-LCLs was measured using a standard 4-h ^51^Cr release assay. Expression of perforin and granzyme B in CD8^+^ T cell lines from the healthy control and XLP patient was also determined (panel [iii]).
(TIF)

Figure S3 Expression of SLAM family receptors on CD8^+^ T cell subsets in XLP carriers. PBMCs from four different XLP carriers were stained with mAb specific for CD8, CD45RA, and CCR7 and either 2B4, NTB-A, CD229, SLAM, CD84, or CRACC; expression of SAP was then detected following fixation and permeabilisation. Expression of each SLAM family member on SAP^−^ and SAP^+^ naïve, central memory, effector memory, and T_EMRA_ CD8^+^ T cells was determined by gating on CD45RA^+^CCR7^+^, CD45RA^−^CCR7^+^, CD45RA^−^CCR7^−^, and CD45RA^+^CCR7^−^ cells, respectively. The graphs show data points (mean fluorescence intensity) for all carriers examined (*n* = 4); the horizontal bar represents the mean.
(TIF)

Figure S4 EBV-specific CD4 T cells are largely SAP^+^. PBMCs from two XLP carriers were either unstimulated or stimulated with EBV lysate or anti-CD3/anti-CD28 mAbs. Expression of IFN-γ by SAP^+^ and SAP^−^ CD4^+^ T cells was determined after 4–6 h. The values represent the proportion of responding cells that were SAP^−^ or SAP^+^.
(TIF)

Figure S5 Increased expression of *SH2D1B* in SAP-deficient XLP memory CD8^+^ T cells. CD8^+^ T cell subsets corresponding to naïve, central memory, effector memory, and T_EMRA_ CD8^+^ T cells were isolated from the peripheral blood of two healthy controls and two XLP patients. Expression of *SH2D1B*, encoding the SAP-related homolog EAT-2, was determined by microarray analysis using Human Genome U133 Plus 2.0 Affymetrix Arrays and GeneSpring software.
(TIF)

## Abbreviations

APC: Ag presenting cell
B-LCL: B-lymphoblastoid cell lines
CMV: cytomegalovirus
CRACC: CD2-like receptor-activating cytotoxic cell
EBV: Epstein Barr virus
Flu: influenza
SAP: SLAM-associated protein
SLAM: signalling lymphocytic activation molecule
T_EMRA_: effector memory cells expressing CD45RA
XLP: X-linked lymphoproliferative disease
