# Supplementary figures and images for: Molecular Pathogenesis of EBV Susceptibility in XLP as Revealed by Analysis of Female Carriers with Heterozygous Expression of SAP
Source: PLoS Biol. 2011 Nov 1;9(11):e1001187. doi: 10.1371/journal.pbio.1001187 (PMC3206011; doi:10.1371/journal.pbio.1001187)

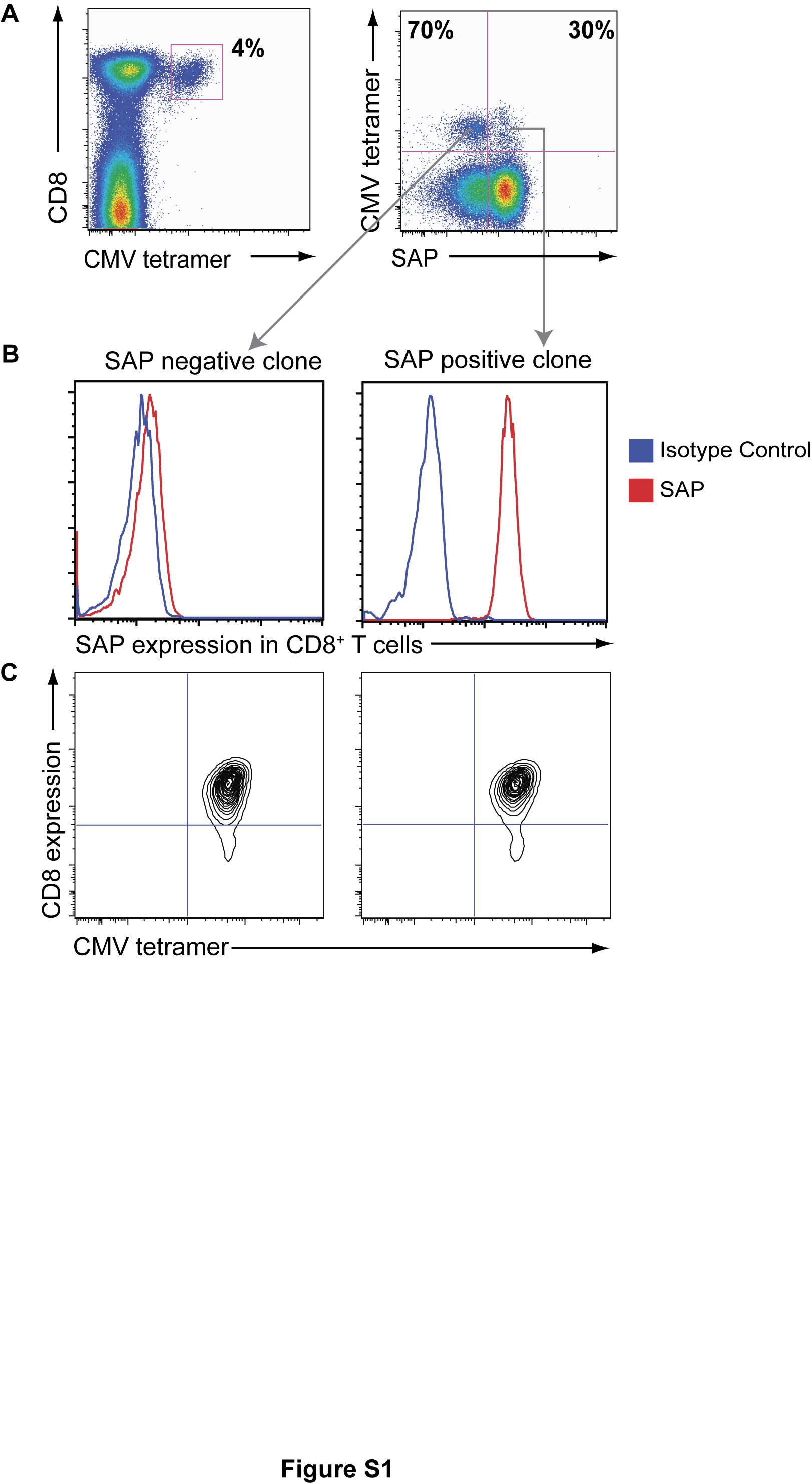

Supplement: Figure S1 — Generation of SAP− and SAP+ virus-specific clones. Virus-specific cells were isolated from PBMCs of XLP carriers by sorting tetramer+ cells (A). Clones were then established by limiting dilution assay and positive clones were expanded. All clones were then examined for their expression of SAP by intracellular staining (B) and specificity by tetramer staining (C). (TIF) [file pbio.1001187.s001.tif]

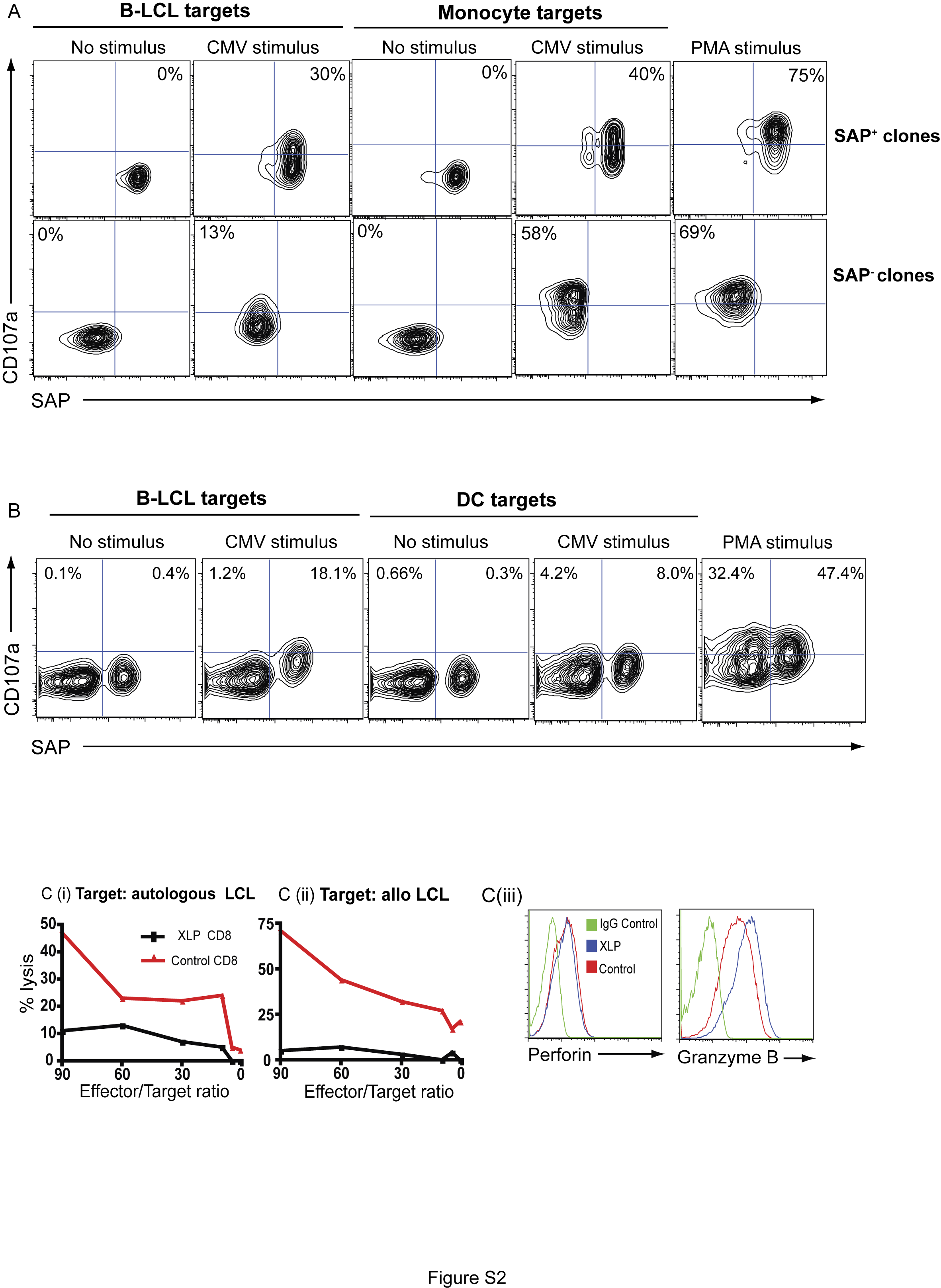

Supplement: Figure S2 — SAP deficient CD8+ T cells fail to respond to B cell targets. (A) Ag-specific SAP+ (upper panel) and SAP− (lower panel) CD8+ T cell clones or (B) EBV-specific CD8+ T cell lines isolated from an XLP carrier were cultured with (A) autologous B-LCLs or HLA-matched monocytes or (B) autologous B-LCLs or HLA-matched DCs that had been pulsed with either an irrelevant or cognate peptide for 4–6 h. Stimulation with PMA/Ionomycin was used as a positive control. Expression of CD107a was then determined. These results are derived from different sets of clones as those presented in Figure 4. (C) EBV-specific CD8+ T cell lines were established from a healthy control or an XLP patient. The ability of these cells to lyse autologous (panel [i]) and allogeneic but HLA-matched (panel [ii]) B-LCLs was measured using a standard 4-h 51Cr release assay. Expression of perforin and granzyme B in CD8+ T cell lines from the healthy control and XLP patient was also determined (panel [iii]). (TIF) [file pbio.1001187.s002.tif]

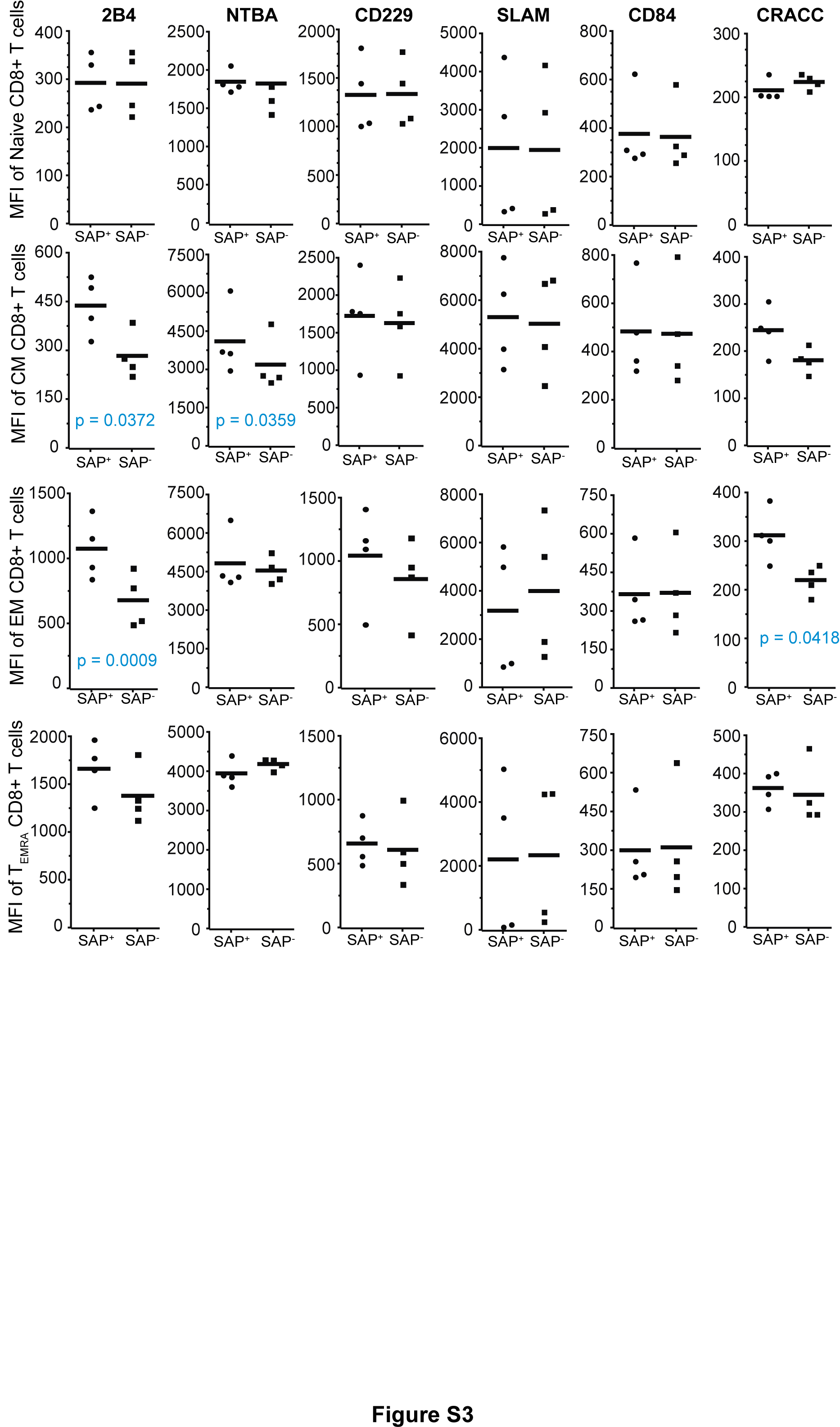

Supplement: Figure S3 — Expression of SLAM family receptors on CD8+ T cell subsets in XLP carriers. PBMCs from four different XLP carriers were stained with mAb specific for CD8, CD45RA, and CCR7 and either 2B4, NTB-A, CD229, SLAM, CD84, or CRACC; expression of SAP was then detected following fixation and permeabilisation. Expression of each SLAM family member on SAP− and SAP+ naïve, central memory, effector memory, and TEMRA CD8+ T cells was determined by gating on CD45RA+CCR7+, CD45RA−CCR7+, CD45RA−CCR7−, and CD45RA+CCR7− cells, respectively. The graphs show data points (mean fluorescence intensity) for all carriers examined (n = 4); the horizontal bar represents the mean. (TIF) [file pbio.1001187.s003.tif]

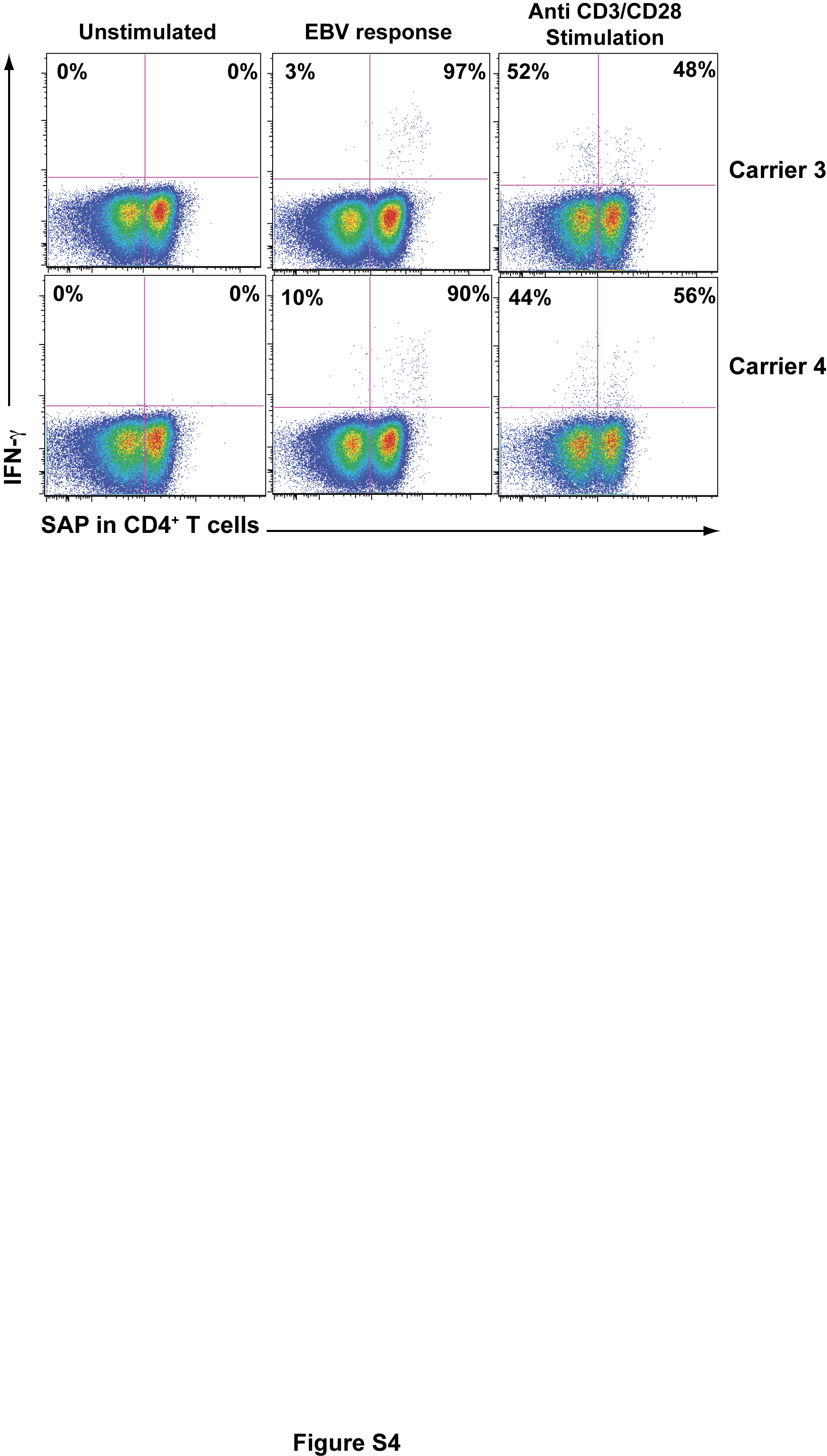

Supplement: Figure S4 — EBV-specific CD4 T cells are largely SAP+. PBMCs from two XLP carriers were either unstimulated or stimulated with EBV lysate or anti-CD3/anti-CD28 mAbs. Expression of IFN-γ by SAP+ and SAP− CD4+ T cells was determined after 4–6 h. The values represent the proportion of responding cells that were SAP− or SAP+. (TIF) [file pbio.1001187.s004.tif]

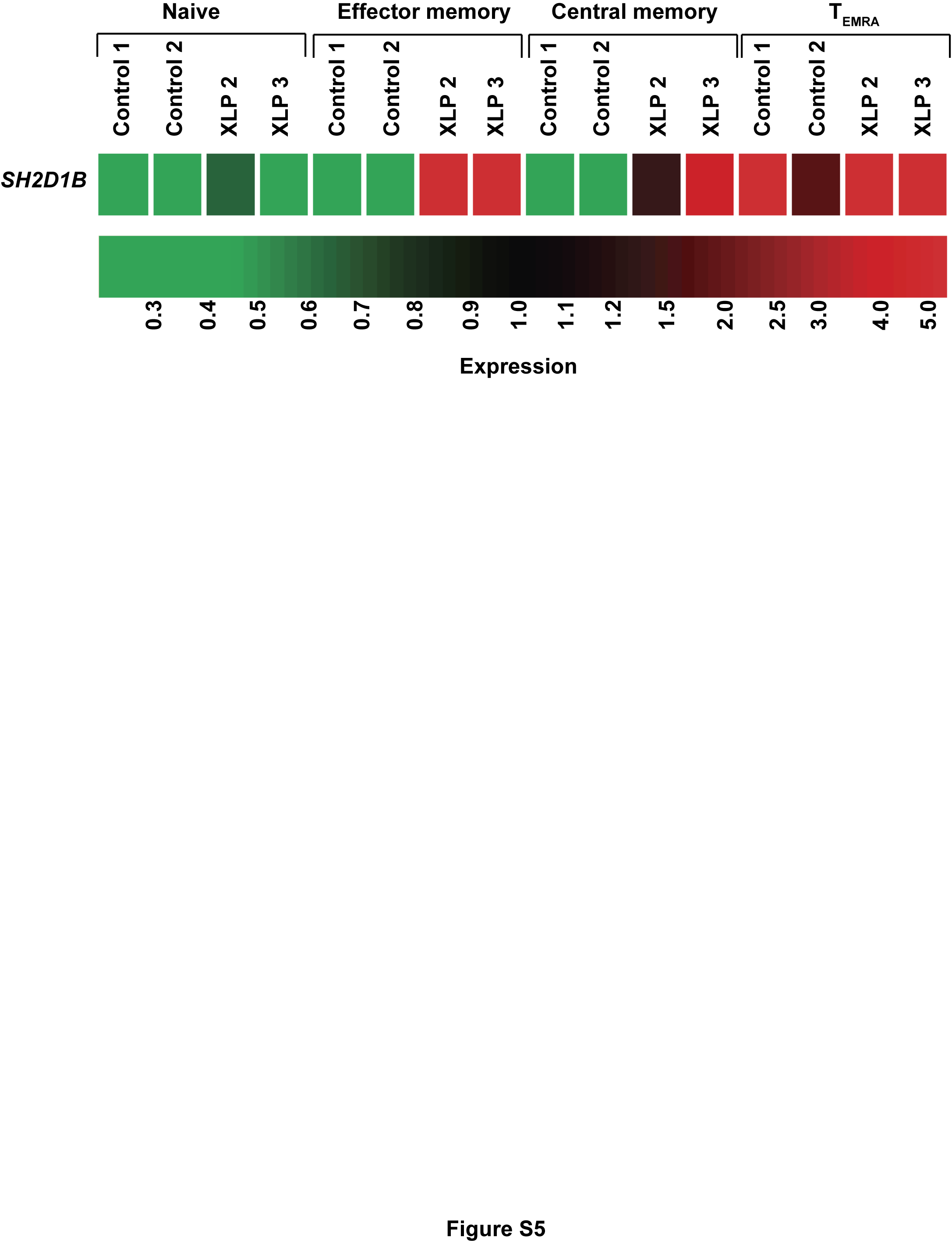

Supplement: Figure S5 — Increased expression of SH2D1B in SAP-deficient XLP memory CD8+ T cells. CD8+ T cell subsets corresponding to naïve, central memory, effector memory, and TEMRA CD8+ T cells were isolated from the peripheral blood of two healthy controls and two XLP patients. Expression of SH2D1B, encoding the SAP-related homolog EAT-2, was determined by microarray analysis using Human Genome U133 Plus 2.0 Affymetrix Arrays and GeneSpring software. (TIF) [file pbio.1001187.s005.tif]
